# Optimized photoluminescence quantum yield in upconversion composites considering the scattering, inner-filter effects, thickness, self-absorption, and temperature

**DOI:** 10.1038/s41598-021-93400-8

**Published:** 2021-07-06

**Authors:** Callum M. S. Jones, Daniel Biner, Stavros Misopoulos, Karl W. Krämer, Jose Marques-Hueso

**Affiliations:** 1 Institute of Sensors, Signals and Systems, Heriot-Watt University, Edinburgh, EH14 4AS UK; 2 Department of Chemistry and Biochemistry, University of Bern, Freiestrasse 3, 3012 Bern, Switzerland; 3 Edinburgh Instruments Ltd., Kirkton Campus, Livingston, UK

**Keywords:** Optical materials and structures, Optical techniques

## Abstract

Optimizing upconversion (UC) composites is challenging as numerous effects influence their unique emission mechanism. Low scattering mediums increase the number of dopants excited, however, high scattering mediums increase the UC efficiency due to its non-linear power dependency. Scattering also leads to greater thermal effects and emission saturation at lower excitation power density (PD). In this work, a photoluminescence quantum yield (PLQY) increase of 270% was observed when hexagonal NaYF_4_:(18%)Yb^3+^,(2%)Er^3+^ phosphor is in air compared to a refractive index-matched medium. Furthermore, the primary inner-filter effect causes a 94% PLQY decrease when the excitation focal point is moved from the front of the phosphor to 8.4 mm deep. Increasing this effect limits the maximum excitation PD, reduces thermal effects, and leads to emission saturation at higher excitation PDs. Additionally, self-absorption decreases the PLQY as the phosphor’s thickness increases from 1 to 9 mm. Finally, in comparison to a cuboid cuvette, a 27% PLQY increase occurs when characterizing the phosphor in a cylindrical cuvette due to a lensing effect of the curved glass, as supported by simulations. Overall, addressing the effects presented in this work is necessary to both maximize UC composite performance as well as report their PLQY more reliably.

## Introduction

Two or more low-energy photons can be converted into a higher energy photon via upconversion (UC). Due to this unique process, UC materials show promise in applications such as, photo-activation processes^1^, bioimaging^2^, biosensing^3^, drug delivery^4^, nanothermometry^5^, and photovoltaics (PV)^6^. Hexagonal sodium yttrium fluoride (β-NaYF_4_) is known to be one of the most efficient host structures for various UC processes^7^. Frequently, Yb^3+^ and Er^3+^ is co-doped into this host to achieve and exploit energy transfer UC, which is the most efficient UC mechanism. The exact concentration of dopants in these structures is particularly important for their performance^8,9^. Unfortunately, due to the forbidden nature of the electronic transitions involved in the UC mechanism and the small absorption coefficients of these materials, the UC efficiency is a significant limitation compared to other photoluminescent materials^10^. Work optimizing the composition of the UC materials, as well as corresponding enhancement techniques^11,12^, is therefore important for increasing their photoluminescence quantum yield (PLQY), which characterizes the efficiency of these materials, and their application potential^13^. The measurement is sub-categorized into the internal PLQY (iPLQY) and external PLQY (ePLQY), depending on whether an appropriate reference sample is or is not used during its determination, respectively^14^. The associated variation in calculation method is detailed in supplementary note 1 of the Supporting Information.

UC materials possess a non-linear dependence on excitation power due to the multi-photon absorption process of UC mechanisms. This adds an additional layer of complexity to PLQY characterization procedures^15^. Unlike conventional methodologies, the PLQY of UC materials depends on the excitation power density (PD)^16^, bandwidth^17^, and beam profile (BP)^18^. Additionally, effects such as excitation beam scattering^19,20^, emission self-absorption^21^, excitation induced thermal effects^22^, and emission around the excitation wavelength^23^, also need to be considered. Furthermore, traditional PLQY methods have explored the impact of the primary inner-filter effect, which is defined as the variations in excitation PD through the sample as the propagating light is absorbed^24,25^. This will also be of consequence in UC characterizations; however, it has received little attention so far. As a result of these influential effects, no PLQY standards are currently available for UC materials^26^. The significance of these effects has left various unanswered questions for achieving maximum performance in UC composites. In response, we present experimental work and simulations to optimize the UC emission and efficiency of β-NaYF_4_:(18%)Yb^3+^,(2%)Er^3+^ composites through optimizing scattering, inner-filter effects, sample geometry, self-absorption, and temperature. Our results yield a deeper understanding of how these effects can be addressed to optimize UC materials and increase reliability in their PLQY measurements. The latter is crucial due to the comparability challenges associated with characterizing UC materials.

## Results and discussion

### The influence of excitation scattering on the observed upconversion emission in the high PD regime (0.025–51.6 W/cm^2^)

**Figure 1:**
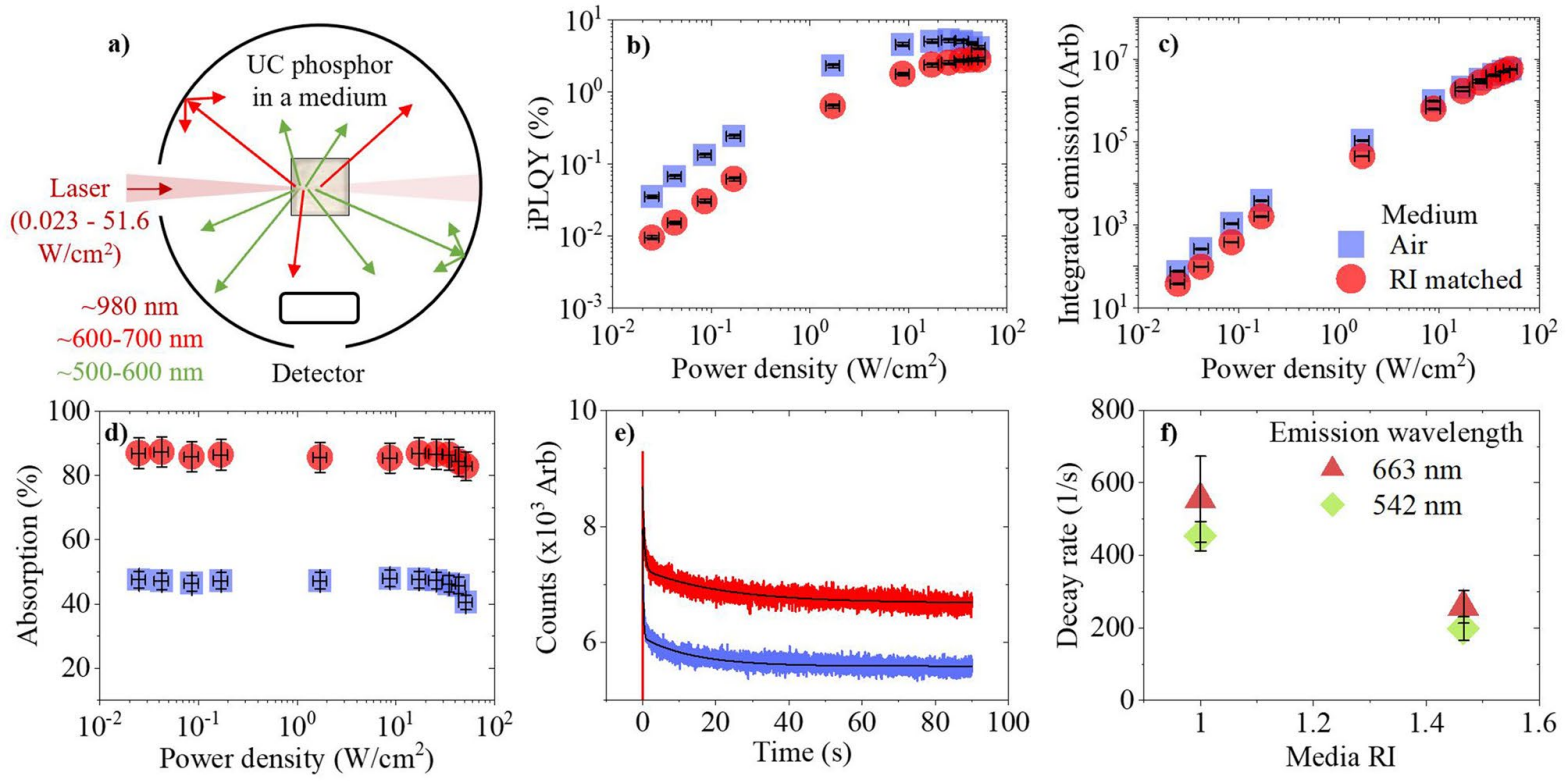
β-NaYF_4_:(18%)Yb^3+^,(2%)Er^3+^ microscale phosphor is placed in air (blue square) or a RI-matched medium (red circle) and excited with a 980 nm laser diode inside an integrating sphere, as depicted in (**a**). The following was obtained against excitation PD: (**b**) iPLQY (500–700 nm), (**c**) integrated emission (500–700 nm), and (**d**) absorption. Excitation induced thermal effects are then investigated to obtain (**e**) the 542 nm emission decay versus excitation time, and (**f**) the 542 nm (rotated green square) and 663 nm (dark red triangle) emission decay rate versus media RI.

The β-NaYF_4_:(18%)Yb^3+^,(2%)Er^3+^ phosphor in air, is compared to an equal weight mixture of the phosphor in a refractive index (RI) matched medium. The RI matched medium with n = 1.466 is achieved through mixing chloroform and toluene; both solvents are miscible and transparent in the near infrared (NIR) spectral region. This RI value is that of NaYF_4_:Yb^3+^,Er^3+^,Tm^3+^ nanocrystals around 980 nm, as reported by Sokolov et al.^27^. As depicted in Fig. 1a, a 980 nm laser diode is focused into the phosphor, which is placed inside an integrating sphere. A small beam profile (BP) is used to achieve a range of excitation values in the high power density (PD) regime (0.025–51.6 W/cm^2^). Scattering effects are seen in the iPLQY (500–700 nm) across the entire power range, as shown in Fig. 1b. When the phosphor is in air, higher iPLQYs are achieved due to local areas of greater PD. At the lowest investigated PD, the iPLQY was increased by 270% compared to the RI-matched medium. However, this disparity decreases as the PD increases; the PD experienced by the sample influences both the rate of emission saturation and the magnitude of excitation induced thermal effects^15,16,28^. iPLQY saturation occurs at lower PDs in a high scattering medium because it experiences a greater local PD because of scattering. This is reflected by the integrated emission results, given in Fig. 1c. For similar phosphor materials it was reported that the red UC emission still increases whilst the green UC emission decreases when saturation in a high PD regime is approached^16^. We observed this decrease in green emission in our work, as shown in Fig. S1(a, b) of the Supporting Information. Although scattering benefits the UC emission intensity in general, eventually these benefits become limited at high PDs; the exact value at which this occurs depends on the specific UC emission. For instance, at 44 W/cm^2^, the RI-matched sample’s green emission becomes greater than the green emission from the higher scattering sample in air. Here, the RI-matched sample experiences a lower PD, smaller thermal effects, and the decrease of the green emission is less. At the same PD, the red emission in air still increases and continues to benefit from scattering. However, we predict that the red emission will show the same behavior when it eventually saturates at higher PD. Finally, the large absorption disparity, as seen in Fig. 1d, gives insight into another effect stemming from increased scattering; beam penetration depth limitation. The more transparent sample allows the excitation beam to propagate deeper into the sample and excite a greater number of dopants, resulting in higher absorption values. In comparison, a significant reduction of ~ 46% is observed when the phosphor is in a high scattering medium. We compare our work to other PLQYs reported for a range of NaYF_4_:Yb^3+^,Er^3+^ phosphor compositions, as shown in Tables S1, S2 in the Supporting Information. It is challenging to make direct comparisons due to the limited data available and the variety of effects that are influencing each reported value differently. However, in our work we observed an iPLQY of 5.16 ± 0.3% (17 ± 3 W/cm^2^) for the green and red UC emissions. This is slightly lower than the 6.8% theoretical value presented by Berry et al.’s group at 25 W/cm^2^, when investigating a similar phosphor composition^29^. However, is does have good comparability with Pokhrel et al., who experimentally observed an ePLQY of 4.16% at 22 ± 0.44 when studying NaYF_4_:(20%)Yb^3+^,(2%)Er^3+^ ^30^.

Thermal effects, induced by the excitation beam, influences the observed PLQY. Investigating these effects also gives insight into the scattering in each sample. The phosphor’s green (542 nm) UC emission decay was measured, as shown in Fig. 1e. Exponential decays are fitted to obtain the decay rates, which are then plotted in Fig. 1f. For both emissions, the intensity decrease in air is faster than in the RI-matched medium. This points to a faster increase of sample temperature in air, as expected for a smaller thermal transmission coefficient. The fitted values used in this investigation, as well as the corresponding red (663 nm) emission decay, are available in supplementary note 4 of the Supporting Information.

### The influence of excitation scattering on upconversion in a low PD regime (3 × 10^–2^ W/cm^2^) and its use as a tool to determine the phosphor’s RI

**Figure 2:**
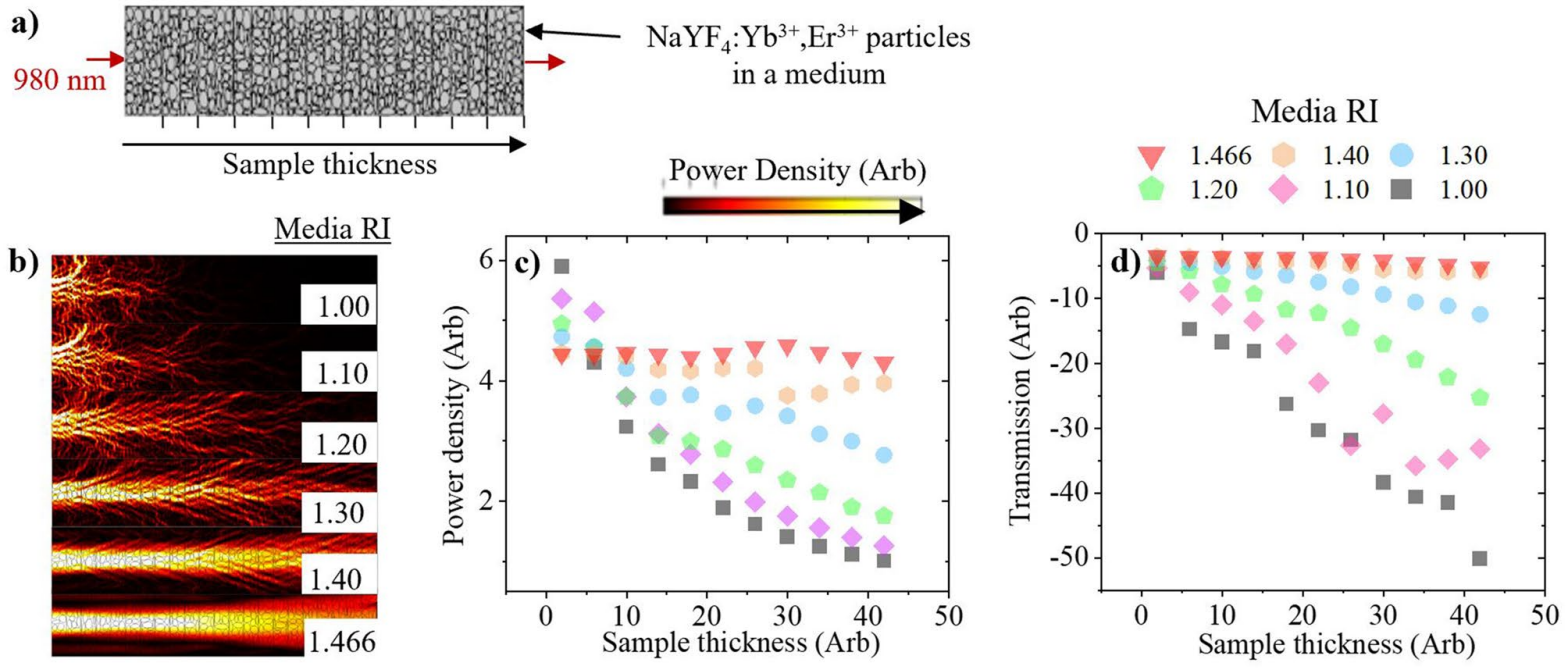
Simulations of a 980 nm beam propagating through powder (n = 1.466) with different sample thicknesses and embedded in a medium of varying RI, as depicted in (**a**) Media RI = 1 (grey square), 1.1 (rotated pink square), 1.2 (green pentagon), 1.3 (blue circle), 1.4 (orange hexagon), and 1.466 (red triangle). At maximum thickness, the simulated PD of various RI media is given in (**b**). The following is measured over the entire sample geometry and presented against sample thickness: (**c**) average PD, and (**d**) incident beam transmission.

In this section, COMSOL Multiphysics is used to simulate the PD and beam transmission when varying the samples thickness and the RI of the surrounding medium. As depicted in Fig. 2a, the sample geometry was modelled from multiple rectangular sections stacked on top of each other, each containing 1–10 μm sized particles with random geometry and n = 1.466. The number of sections varies depending on the thickness under investigation. Electromagnetic radiation, of 980 nm, is generated at the bottom of the first section and is absorbed at the top of the final section. Details on the simulation geometry and associated parameters used are given in supplementary note 5 of the Supporting Information. Select simulations of the thickest samples are shown in Fig. 2b, where it is seen that scattering is most prominent towards the front of the sample. At small sample thickness, an increasing RI disparity leads to higher PDs, as shown in Fig. 2c. The PD decreases as the RI of the medium increases towards n = 1.466, since the sample becomes more transparent. In the RI-matched medium, the average PD is similar irrespective of the sample thickness because minimal light is scattered away from the forward direction. This is supported by Fig. 2d, which shows a constantly high transmission at each sample thickness. The transmission decreases as the RI of the medium approaches n = 1 due to the greater amounts of scattering.

**Figure 3:**
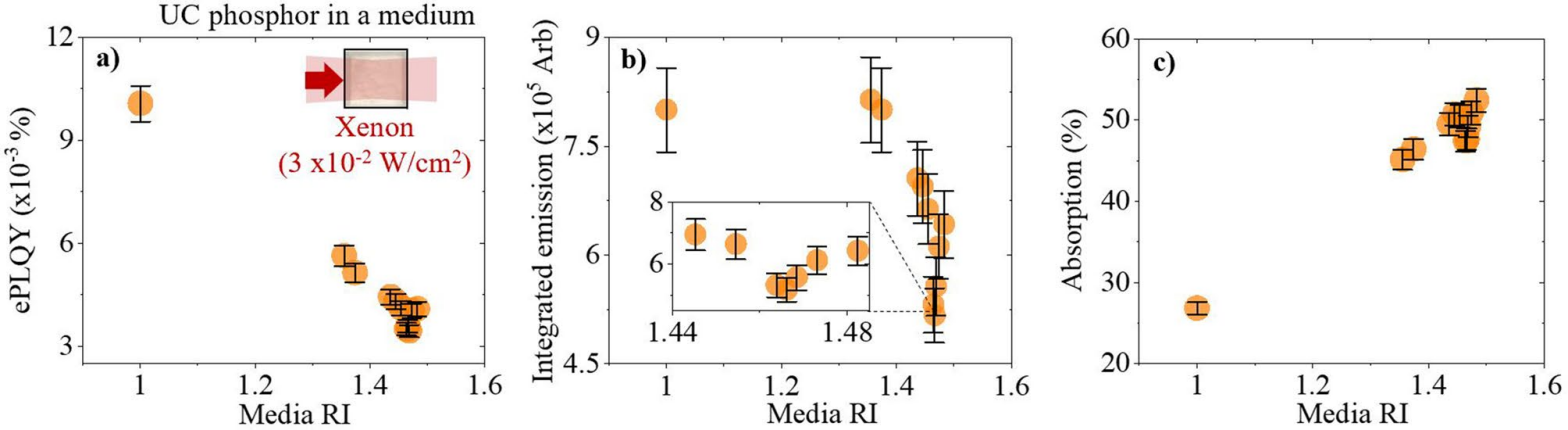
β-NaYF_4_:(18%)Yb^3+^,(2%)Er^3+^ phosphor is placed in air or a transparent medium with varying RI and excited with a 980 nm Xe lamp (orange circle). The following was measured against media RI: (**a**) the ePLQY (500–700 nm), with an inset depiction of the excitation conditions inside each sample, (**b**) the integrated emission (500–700 nm) with an inset magnification of the data points between n = 1.44–1.485, and (**c**) the absorption.

The simulations highlight that a range of scattering regimes could be obtained by embedding the UC phosphor in mediums of varying RI. By using known RI media, scattering can be a tool to experimentally estimate the RI of the β-NaYF_4_:(18%)Yb^3+^,(2%)Er^3+^ phosphor. A Xe lamp possessing a large BP and centred at 980 nm is used as an excitation source in the low PD regime. The phosphor is embedded in range of media with 1 < n < 1.5, which are transparent to the NIR. As seen in Fig. 3a, the ePLQY scales with RI disparity; it is highest for n = 1 and lowest for n = 1.466, when the sample is most transparent. The determined phosphor RI of n =  ~1.466 is similar to the value previously reported^27^. From RI-matched medium to air, the ePLQY (500–700 nm) increases by 181%. The integrated emission, seen in Fig. 3b, follows a similar trend; the smallest intensity is observed when the sample is most transparent. However, the influence of the competing beam penetration depth effect can be observed in the highest scattering regimes. Less dopants are excited when there is a large RI disparity, which limits the integrated emission despite the benefits to PD. The absorption, shown in Fig. 3c, is smallest for the lowest RI medium, which corresponds to the high scattering regime. The absorption values increase as scattering decreases, up to a point where changes become small when the RI disparity is low. The associated emission spectra are shown in Fig. S4 of the Supporting Information. Both the green and red UC emissions follow the same trend because thermal and saturation effects are negligible in this PD regime.

### The influence of the primary inner-filter effect on the observed upconversion in a high PD regime (0.169 W/cm^2^)

**Figure 4:**
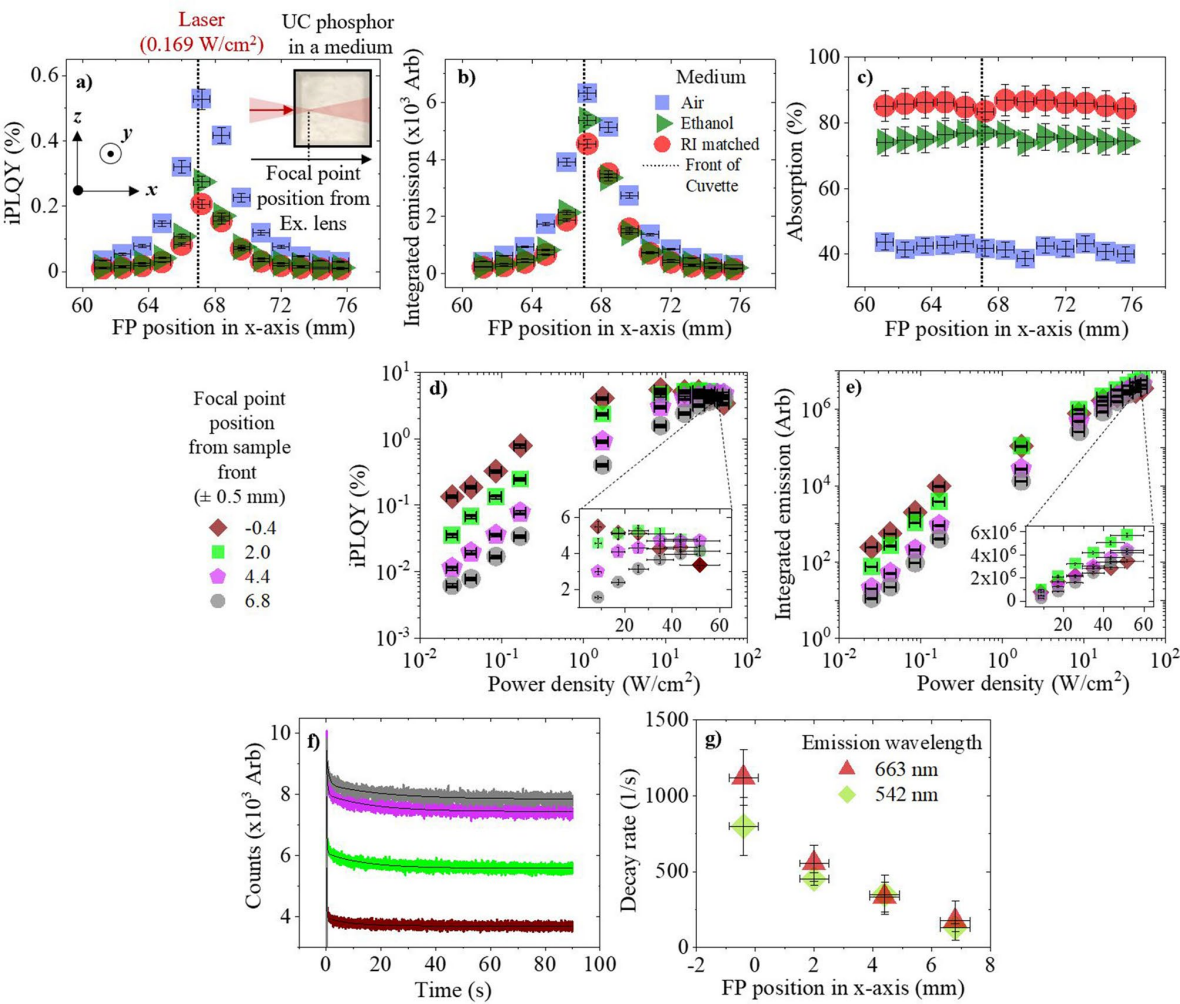
β-NaYF_4_:(18%)Yb^3+^,(2%)Er^3+^ phosphor is excited with a 980 nm laser diode, the FP of which, is moved through the sample in the x-axis. The phosphor is air (blue square), ethanol (dark green triangle), or a RI matched medium (red circles). The following is measured against the FP position from the excitation lens: (**a**) iPLQY (500–700 nm), with an inset depiction of the excitation conditions inside each sample, (**b**) integrated emission (500–700 nm), and (**c**) absorption. The second experiment investigates moving a high PD regime, where the FP is moved to four positions: − 0.4 (rotated brown square), 2 (light green square), 4.4 (pink pentagon), and 6.8 ± 0.5 mm (grey circle), relative to the front of the cuvette. At various FP positions through the sample, the following is presented against excitation PD: (**d**) iPLQY (500–700 nm), with an inset magnification of results at the highest PDs, and (**e**) the integrated emission (500–700 nm), with an inset magnification of the results at the highest PDs. The third experiment investigates excitation induced thermal effects at various FP positions. At various FP positions through the sample, the following is obtained: (**f**) the 542 nm emission decay versus excitation time, and (**g**) the 542 nm (rotated green square) and 663 nm (dark red triangle) emission decay rate versus FP position.

The primary inner-filter effect is another aspect that should be considered when characterizing UC phosphors as it limits the maximum PD of the focal point (FP), which is where the majority of UC is generated. Related to this, the scattering of light away from the areas of highest PD will also be a limitation. Both these effects increase with the length of the beam path in the phosphor material. To test this, the FP from a 980 nm excitation laser diode is moved in the x-axis through the β-NaYF_4_:(18%)Yb^3+^,(2%)Er^3+^ phosphor, as depicted in the inset of Fig. 4a. An equal weight of phosphor is tested in air, as well as ethanol and a RI matched medium. Figure 4a shows the iPLQY of the phosphor at various FP positions, measured from the excitation lens. By placing the FP at the front of the sample, the highest iPLQY is achieved. If the FP is placed before the sample, the iPLQY decreases since the beam diverges, reducing the PD incident on the UC material. Due to the primary inner-filter effect, the iPLQY decreases by 21%, 77%, 91%, and 94% when the FP moves 1.2, 3.6, 6, and 8.4 mm into the sample, respectively. Similar trends were observed for the phosphor dispersed in media however, the iPLQY values are reduced as the samples RI disparity decreases due to scattering. Compared to air, the maximal iPLQY at the front of the sample decreases by 48% in ethanol and 61% in the RI-matched medium. Corresponding findings are observed in the integrated emission, given in Fig. 4b, but not the absorption, as displayed in Fig. 4c. The absorption remains constant despite the variation in FP position. This is significant as it highlights that the excitation is absorbed equally in each case despite differences in the experienced PD. The related emission spectra are provided in the Supporting Information (Fig. S5). Thermal and saturation effects are relatively small and therefore no obvious differences are observed between the green and red UC emissions. Moving the FP in the y-axis (vertically) has negligible effects since the primary inner-filter effect remains constant, as shown in Fig. S6 of the Supporting Information.

The primary inner filter effect is then investigated in a high PD regime (0.025–51.6 W/cm^2^). The phosphor is placed in air and the excitation FP is moved to four different x-axis positions in the phosphor. As seen in Fig. 4d, the iPLQY decreases by 96% when the FP is moved 6.8 mm into the sample at 0.025 W/cm^2^. The differences in iPLQY decrease for higher excitation PD because the samples experience different PDs, which leads to the UC emission saturating at different rates. This is seen in Fig. 4e as the ratio between the integrated emission at each FP position changes as the excitation PD increases. When the FP is at the front of the sample, the sample experiences the highest PD. Therefore, saturation effects have greater influence on the iPLQY at lower PDs. The related emission spectra are provided in Figs. S7(a, b) of the Supporting Information. The FP position effects are more prominent in the green UC emission, as it saturates at lower PDs compared to the red UC emission. The green net emission at the deepest FP overtakes the net emission at the shallowest FP when the excitation PD is sufficiently high enough. This is because a deeper FP requires a greater excitation PD to achieve emission saturation. This is not as pronounced for the red emission because it saturates at higher PDs, out of the investigation range.

To support these claims, excitation induced thermal effects are investigated at various x-axis FP positions. The 542 nm emission decay with respect to excitation time is displayed in Fig. 4f. Exponential decays are fitted and the decay rates are determined. The decay rates are plotted in Fig. 4g, where they are observed to increase as the excitation FP is moved towards the front of the sample. This is a result of the higher PD experienced by the sample as the primary inner-filter effect is minimized. The fitted values used in this investigation, as well as the corresponding red (663 nm) emission decay, are available in supplementary note 10 of the Supporting Information. Overall, unlike conventional PLQY methods, the FP position must be optimized during UC PLQY characterization to reduce over- and under-estimations of the materials efficiency at a given PD.

### The influence of sample geometry on the observed upconversion in a low PD regime (3 × 10^–2^ W/cm^2^)

**Figure 5:**
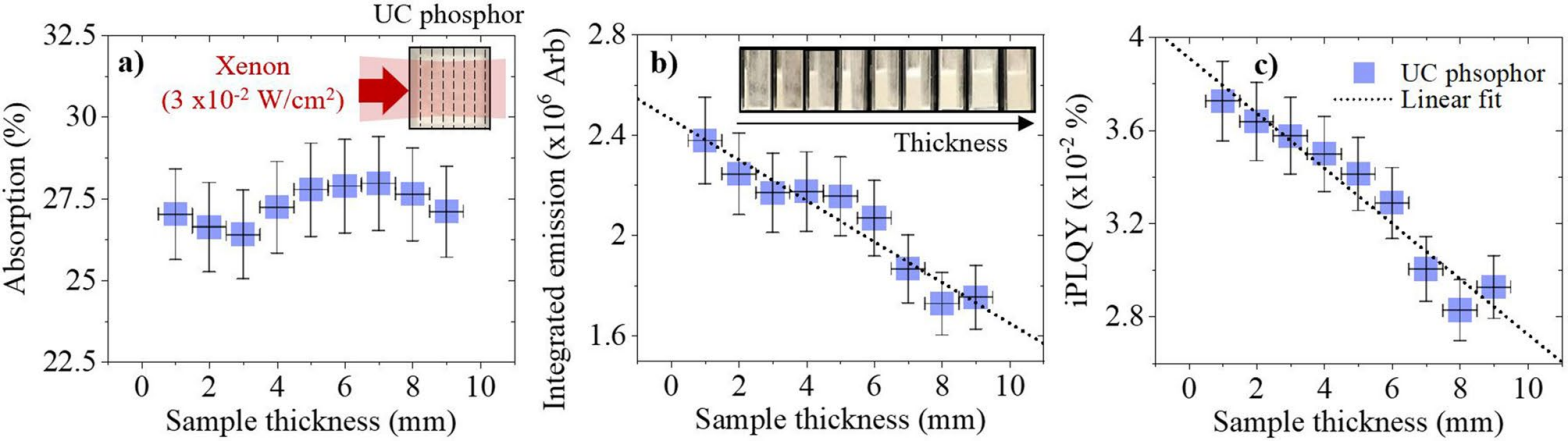
β-NaYF_4_:(18%)Yb^3+^,(2%)Er^3+^ phosphor is measured at various sample thicknesses using 980 nm Xe lamp excitation (blue square). The following is presented against sample thickness: (**a**) absorption, with an inset depiction of the excitation conditions inside each sample, (**b**) integrated emission (500–700 nm) with inset pictures of each sample’s side-profile, and (**c**) iPLQY (500–700 nm).

Currently, many experimental variations exist when preparing an UC sample for PLQY analysis. For instance, various samples have been pressed into a thin layer^31,32^, others have been placed in cylindrical cuvettes or holders^9,32–34^, whilst others are embedded in cuboid quartz cuvettes^35,36^. Moreover, these details are frequently absent, as seen in Tables S1, S2 in the Supporting Information. Sample thickness is investigated here using a Xe lamp with a large excitation BP. The β-NaYF_4_:(18%)Yb^3+^,(2%)Er^3+^ phosphor with layer thicknesses from 1 to 9 mm is prepared using a glass wall separator, as pictured in the inset of Fig. 5a. The samples are placed perpendicular to the excitation beam inside the integrating sphere to obtain the most realistic comparison between the different UC samples. A perpendicular beam minimizes beam spot elongation and spatial energy distribution variations that become more prominent with an inclined beam. This detail has minimal consequences for materials with a linear power dependency; however as already stated, UC materials are very sensitive to excitation BP and PD distortions. In Fig. S9 of the Supporting Information, we show how these distortions occur when rotating the UC phosphor and picture the UC emission emerging from a more oval shaped region when it is non-perpendicular to the excitation beam. Unfortunately, placing the sample perpendicular to the excitation beam can increase backscatter and light loss through the integrating sphere port, which is referred to as “light leakage”^37,38^. This problem will be addressed in the following paragraphs.

As Fig. 5a shows, no significant deviation in absorption is observed for different sample thicknesses. This suggests that the majority of NIR light is absorbed in the first 1 mm of the phosphor. As such, we can also state that the sample generates minimal UC in the volume behind the first 1 mm of phosphor. This compliments with Fig. 5b, where the integrated emission decreases as sample thickness increases, despite additional dopants in the excitation beam path. A decrease of 26% occurs between 1 and 9 mm thick samples, which is similar to the 21% decrease of UC iPLQY displayed in Fig. 5c. Data regarding the linear fits and the related emission spectra are given in supplementary note 12 of the Supporting Information. The emission spectra highlights that these limitations occur for both the green and red UC emissions. The decrease of UC emission for thicker samples is due to two factors: (1) emission self-absorption, and (2) light leakage due to back-scattering. The former is significant because as sample thickness increases, the emission propagates through a longer path in the sample, which increases the probability of self-absorption by the sample^21^. Conventional PLQY correction methods have been established to amend for this type of effect^39,40^, however, these are not appropriate for non-linear UC excitation mechanisms. An estimate of the self-absorption is achieved here by comparing the absorption of doped and undoped samples in the visible range. Doped and undoped samples, both 9 mm thick, were measured in the integrating sphere at 540 nm and 660 nm, as shown in Fig. S11 of the Supporting Information. The scattering in both samples is similar. Therefore, the difference between their spectra represents the absorption by the doped sample. A value of 11.5% is estimated for 500–700 nm, which appears reasonable for the 2% Er^3+^ doped sample. The second effect, light leakage, is regarded as an experimental limitation. Its probability increases with sample thicknesses due to additional scattering centres. To obtain an estimate for light leakage, a 9 mm thick undoped NaYF_4_ sample and an empty cuvette are measured at 540 nm and 660 nm, as shown in Fig. S12 of the Supporting Information. A difference of 21% between these measurements indicates the loss of visible radiation (500–700 nm) due to light leakage. A small amount of UC is also lost by direct propagation through the excitation port after it is generated. However, the variation of this effect is thought to be minimal since a similar number of dopants are excited in each sample, and excitation occurs at comparable locations within the first 1 mm of the sample.

The ratio between sample thickness and doping concentration can be optimized to minimize self-absorption effects, as described by Boccolini et al.^21^. However, if scattering is exploited for additional UC benefits, this relationship will be significantly altered. Since the effects of scattering are most prominent within the first 1 mm of the sample, we hypothesize that it will push the optimal sample thickness to smaller values compared to a semitransparent counterpart. Furthermore, we conclude that self-absorption reduces the reliability of the UC PLQY measurement. The sample thickness should be optimized for the UC PLQY and reported alongside its value. UC PLQY reliability also suffers due to light leakage. Therefore, the excitation and emission port sizes of the integration sphere should be minimized to reduce this effect, whilst also keeping the sample and beam perpendicular to reduce distortions in the BP and PD.

### The influence of cuvette type on the observed upconversion in a high PD regime (0.169 W/cm^2^)

**Figure 6:**
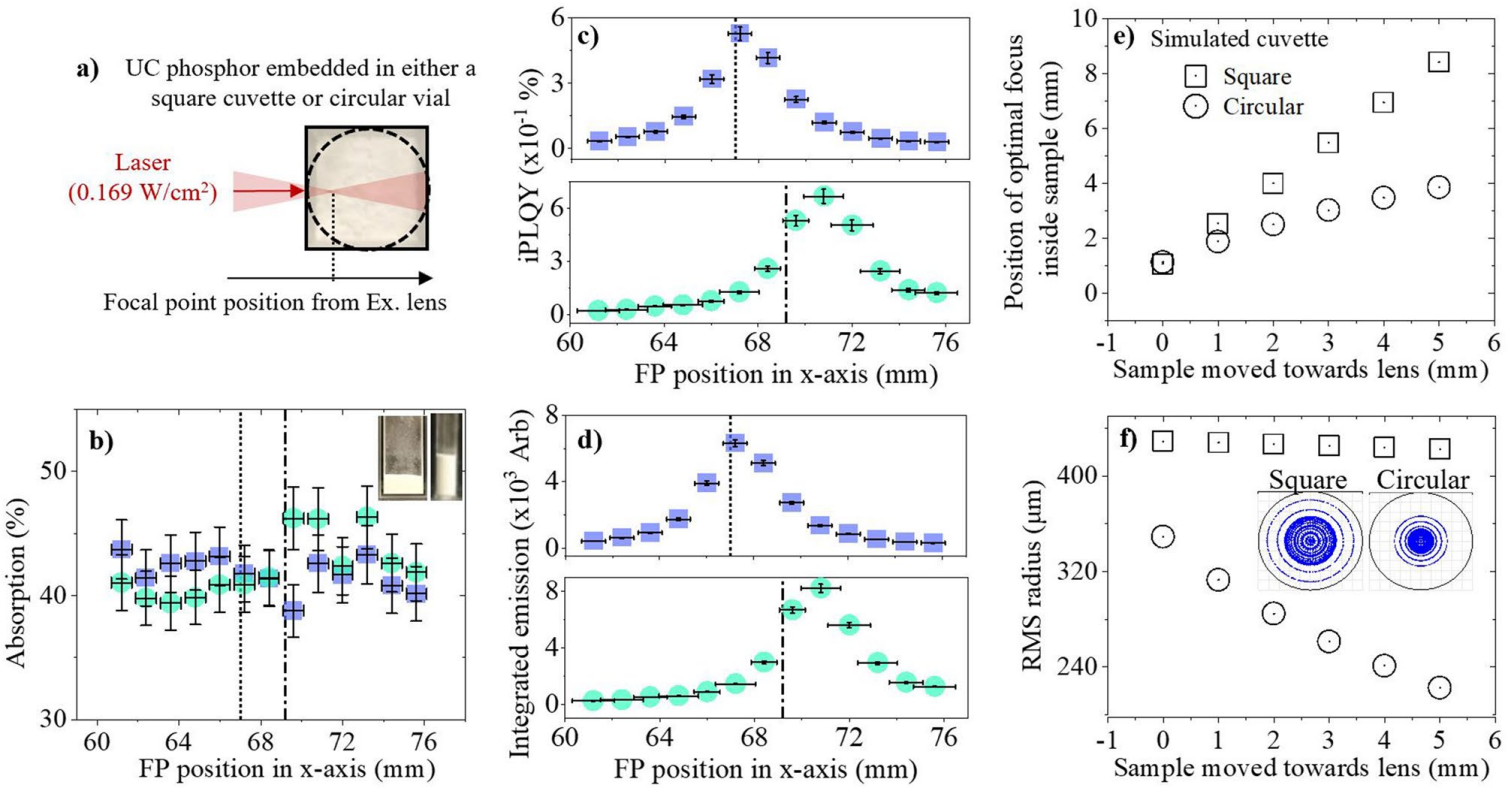
β-NaYF_4_:(18%)Yb^3+^,(2%)Er^3+^ phosphor is embedded in either a cuboid cuvette (blue square) or a cylindrical vial (light blue circle), and excited using a 980 nm laser diode, as shown in (**a**). The following is presented against the lasers excitation FP positions: (**b**) absorption, (**c**) iPLQY (500–700 nm), with inset images of the cuboid and cylindrical containers used, and (**d**) integrated emission (500–700 nm). The second investigation simulates square and circular containers moving towards the excitation lens to represent different FP penetration depths through the sample. The following is measured against sample position: (**e**) position of optimal focus inside container, and (**f**) RMS radius, with an inset image of the simulated beam FP in a square cuvette (left) and circular vial (right) when each sample is at its closest simulated position to the excitation lens.

The significance of sample thickness on the UC emission indicates that similar geometries should be used for reliable PLQY comparison. The sample cuvette should also be similar for good comparability. To investigate the effect of this, a cuboid cuvette (10 × 10 mm) and a cylindrical vial (0.6 mm diameter) are compared. The samples are characterized at various excitation FP positions using a 980 nm laser diode to ensure that the maximum UC is achieved and inner-filter effects are minimal, as shown in Fig. 6a. Figure 6b highlights that the absorption is similar across all FP positions for each sample despite their differences. On the other hand, the UC iPLQY and integrated emission are slightly higher in the cylindrical vial sample, as seen in Fig. 6c,d. Compared to the cuboid cuvette, the maximum iPLQY, which occurs at the front of each sample, is 27% greater. As shown in Fig. S13 of the Supporting Information, both the green and red UC emission spectra are increased. Since the absorption is constant and a similar phosphor weight was embedded in each container, the PLQY differences likely occur due to a variation in excitation conditions. We propose that the UC benefit occurs due to a perturbation of the excitation FP caused by the curved glass of the cylindrical vial.

To further support this assumption, 2D Zemax™ simulations are presented. The position and size of the spot from a focused 980 nm beam is simulated after it propagates through a sample in either a square cuvette or circular vial. Each sample is moved towards to the excitation lens in 1 mm intervals, which represents the laser being focused deeper into the sample. Additional simulation data regarding this investigation can be found in supplementary note 16 of the Supporting Information. Figure 6e relates the position of optimal focus in the sample as it moves closer to the excitation lens. Although the FP locations are initially similar between both samples, a disparity grows as they move closer to the excitation lens. The curved glass of the circular vial sample focusses the light, which keeps the optimal focal position closer to the front of the sample compared to the square cuvette sample. Theoretically, this would be beneficial to UC as the primary inner-filter effect is less significant towards the front of the sample. Furthermore, Fig. 6f showcases the decrease in beams root mean square (RMS) radius that occurs in the circular vial as the sample moves towards the excitation lens and the lensing effect curves the light over a greater distance. Comparatively, the RMS radius is larger in the square cuvette sample and remains constant at each sample position. Smaller RMS radius values would benefit UC as the sample experiences a smaller BP and therefore a greater PD at the FP. The inset picture of simulated BPs in Fig. 6f highlights that when the beam is at its deepest penetration depth, the power is more focused towards the center of the FP in the cylindrical vial compared to the square cuvette. Overall, UC PLQYs can be compared with higher reliability if similar sample cuvettes are used during characterization. Although higher UC occurs in a cylindrical vial, a cuboid cuvette is more ideal for reliable PLQY measurements to minimize uncertainties in the excitation PD.

## Conclusion

Overall, various effects significantly influence the observed PLQY of UC materials, as shown using β-NaYF_4_:(18%)Yb^3+^,(2%)Er^3+^ phosphor. Addressing them is a requirement for fully maximising the performance of UC composites and increasing their application potential, as well as reporting their PLQY with higher comparability.

Our investigations into scattering highlight that UC emission and PLQY benefits are present over a broad range of excitation PDs. This occurs because scattering increases the PD experienced by the sample, which benefits UC due to its non-linear power dependency. At 0.025 W/cm^2^, a 270% increase in ePLQY occurs when the phosphor is in air compared to a RI-matched medium. High scattering also causes the emission to saturate at lower excitation PDs as well as greater thermal effects. Significantly lower absorption values are measured in comparison to the RI-matched sample, which indicates that scattering also limits the beam penetration depth. COMSOL simulations show the effects of scattering for various RI media and sample thicknesses, when exciting with a broad FP. This data provides theoretical evidence that scattering increases the PD within the sample, whilst also limiting the beam penetration depth. It also helps to show that scattering is most beneficial at the very front of the sample. By using a Xe lamp with a broad excitation, we exploit the UC emission sensitivity to scattering as a tool to estimate the β-NaYF_4_:(18%)Yb^3+^,(2%)Er^3+^ RI to be n =  ~1.466, by embedding it in mediums of known RI. The UC ePLQY scales with RI disparity. The integrated emission also increases with RI disparity, however, the reduction in beam penetration depth limits the integrated emission at very large RI disparities (Δn > 0.355). This limitation also leads to a reduction in sample absorption as scattering increases. UC phosphors are commonly characterised in air, which creates a high scattering medium. Therefore, reported UC PLQYs commonly overestimate the UC efficiencies available at given excitation PDs due to scattering effects. The data implies that maximising transparency and absorption in UC composites, which is common for applications, will not always yield optimal UC performance.

When varying the excitation FP position in the x-axis through the phosphor, the primary inner-filter effect and back-scattering limit the excitation PD reaching the FP. Compared to the front of the sample, the iPLQY decreases by 21%, 77%, 91%, and 94%, when the FP moves 1.2, 3.6, 6, and 8.4 mm into the sample, respectively. The absorption remains constant at each FP position. Increasing the primary inner-filter effect also leads to UC emission saturation at lower excitation PDs, as well as higher thermal effects. Therefore, there is a requirement to optimise the excitation FP position, by placing it at the very front of the sample, to maximise the performance of UC composites as well as reduce UC PLQY discrepancies.

The phosphor geometry is proven to have effects on the UC emission. The UC integrated emission and iPLQY decrease respectively by 26% and 21% when the sample thickness is increased from 1 to 9 mm. The absorption remains constant irrespective of sample thickness, suggesting that the majority of UC emission occurs in the first 1 mm of the phosphor. In terms of the thickest sample, it is estimated that self-absorption accounts for 11.5% of total emission loss and light leakage due to backscattering contributes 21%.

Finally, the phosphor has higher UC emissions and iPLQYs when embedded in a cylindrical vial instead of a cuboid cuvette. Using Zemax™ simulations, it is proposed that this is due to a lensing effect introduced by the curved glass on the cylindrical vial. This decreases the RMS of the laser beam’s spot, increasing the excitation PD at the FP. Additionally, the spot moves closer to the front of the sample, which reduces the primary inner-filter effect. Both effects are beneficial for enhancing UC.

Future work involves applying the findings in this work to optimise UC composites for application purposes. For example, UC-PV devices could benefit greatly from introducing scattering. Furthermore, future PLQY studies can benefit from adjusting their experimental and reporting methodology to address the effects highlighted in this work. For instance, the following is stated: (1) work that characterises UC phosphors in air should acknowledge that scattering is significantly influencing the stated PLQY and associated PD values. (2) Although characterising UC phosphors in a standard 10 × 10 mm cuboid quartz cuvette minimising FP distortions and PD uncertainties, the value obtained will not represent the optimal PLQY due to effects such as self-absorption. The PLQY should be characterised for an optimised sample thickness, which is then stated. Work has been done in regard to finding such optimal thicknesses^21^, however, we note that this thickness will likely change in a high scattering medium. (3) Reducing the primary inner filter effect is crucial for increasing PLQY comparability at a given excitation PD. This is achieved by optimizing the excitation FP position to the front of the UC sample prior to characterisation.

## Methods

### Materials

Samples of phase pure β-NaYF_4_ and β-NaYF_4_:(18%)Yb^3+^,(2%)Er^3+^ were synthesized following a previously published procedure^7^, additional details are given in supplementary note 17 of the Supporting Information.

### Characterization

An FLS920 spectrofluorometer (Edinburgh Instruments) and an integration sphere (Yobin Yvon), with a 102 mm inner diameter, was used for all UC PLQY investigations. The sample was placed in the center of the integrating sphere and an extended red-sensitive photon multiplier (Hamamatsu, R2658P) detector was used to record all spectra. Additional details on the excitation sources and methodology specific to each PLQY study, as well as the emission decay measurements, are given in supplementary note 18 of the Supporting Information.

## Supplementary information

Supplementary Information.
