# Supplementary material for: Optimized photoluminescence quantum yield in upconversion composites considering the scattering, inner-filter effects, thickness, self-absorption, and temperature
Source: Sci Rep. 2021 Jul 6;11:13910. doi: 10.1038/s41598-021-93400-8 (PMC8260772; doi:10.1038/s41598-021-93400-8)
Supplement: Supplementary file 1 — Supplementary Information. [file 41598_2021_93400_MOESM1_ESM.pdf]

Supporting Information

Optimized photoluminescence quantum yield in upconversion composites considering the scattering, inner-filter effects, thickness, self-absorption, and temperature

Callum M. S. Jones, [1] Daniel Biner, [2] Stavros Misopoulos, [1,3] Karl W. Krämer, [2] Jose Marques-Hueso,*,[1]

[1]Institute of Sensors, Signals and Systems, Heriot-Watt University, Edinburgh, EH14 4AS, United Kingdom
[2]University of Bern, Department of Chemistry and Biochemistry, Freiestrasse 3, 3012 Bern, Switzerland.
[3]Edinburgh Instruments Ltd., Kirkton Campus, Livingston, United Kingdom
*J. M.H.: email, j.marques@hw.ac.uk

Contents (Supplementary note) **Page**

1. Calculating the PLQY 2
2. Emission spectra relating to the investigation into excitation scattering on the observed upconversion in a high PD regime (0.025 – 51.6 W/cm^2) 2
3. Comparing reported $NaYF_4$:Yb^{3+},Er^{3+} microscale phosphor PLQYs using review tables 3
4. Fitted values used in the investigation of thermal effects in different scattering media and the 663 nm UC emission decay........ 6
5. Simulation parameters and geometry used in COMSOL scattering investigations 7
6. Emission spectra relating to the investigation into the influence of excitation scattering on upconversion in a low PD regime (3 x10^{-2} W/cm^2) 8
7. Emission spectra relating to the investigation into the influence of the primary inner-filter effect on the observed upconversion in a high PD regime (0.169 W/cm^2)........ 8
8. PLQY investigation into the influence of moving the excitation FP in the y-axis (vertically) whilst inside the sample, conducted in a high PD regime (0.169 W/cm^2)........ 9
9. Emission spectra relating to the investigation into the primary inner-filter effect on the observed upconversion in a high PD regime (0.025 – 51.6 W/cm^2)........ 10
10. Fitted values used in the investigation of thermal effects at various excitation FP positions in the x-axis and the 663 nm UC emission decay........ 11
11. Evidence of excitation beam distortion due to exciting samples at an angle........ 12
12. Emission spectra relating to the investigation into sample thickness on the observed upconversion in a low PD regime (3 x10^{-2} W/cm^2) 13
13. Estimating the magnitude of UC emission self-absorption. 14
14. Estimating the magnitude of UC emission loss due to back-scattering (light leakage)........ 14
15. Emission spectra relating to the investigation into the influence of cuvette type on the observed upconversion in a high PD regime (0.169 W/cm^2)........ 15
16. Simulation parameters and geometry used in Zemax investigations........ 16
17. Synthesis of $NaYF_4$:(18%)Yb^{3+},(2%)Er^{3+} UC phosphor 17
18. Experimental characterisation methodology (PLQY and thermal investigations) 18

References 19

Supplementary note 1: Calculating the PLQY

The two-measurement method (2MM) [1], as well as the three-measurement method (3MM) are commonly used for absolute UC PLQY characterizations [2]. The 2MM method was selected for this work because several studies have shown that it yields results with very good agreement to the 3MM method, and distinguishing between the two was unnecessary [3-5]. Equation (1) defines the PLQY, also referred to as the internal PLQY (iPLQY), which is calculated by dividing the emission intensity (L_{Sample}) by the number of absorbed photons. Here, $E_{Reference}$ and E_{Sample} represent the excitation light intensity not absorbed by a reference or by the UC sample, respectively. Here, the iPLQY represents the efficiency of the UC mechanism by considering the scattering and absorption effects of a non-emitting reference (typically the undoped host) that is similar in size and scattering coefficient to the sample under investigation. Alternatively, the external PLQY (ePLQY) can be determined when no sample is used as a reference. As such, instead of the number of absorbed photons, the number of incident photons is measured. This can be useful for characterizing the entire material under investigation and not just the lanthanide dopants.

$$\text{PLQY} = \frac{\text{\# photons emitted}}{\text{\# absorbed photons}} = \frac{L_{Sample}}{E_{Reference} - E_{Sample}} \tag{1}$$

Supplementary note 2: Emission spectra relating to the investigation into excitation scattering on the observed upconversion in a high PD regime (0.025 – 51.6 W/cm^2).

Hexagonal $NaYF_4$:(18%)Yb^{3+},(2%)Er^{3+} UC phosphor in air and a RI-matched medium are compared via a PLQY investigation. Fig. S1 displays the corresponding emission spectra obtained for this investigation.

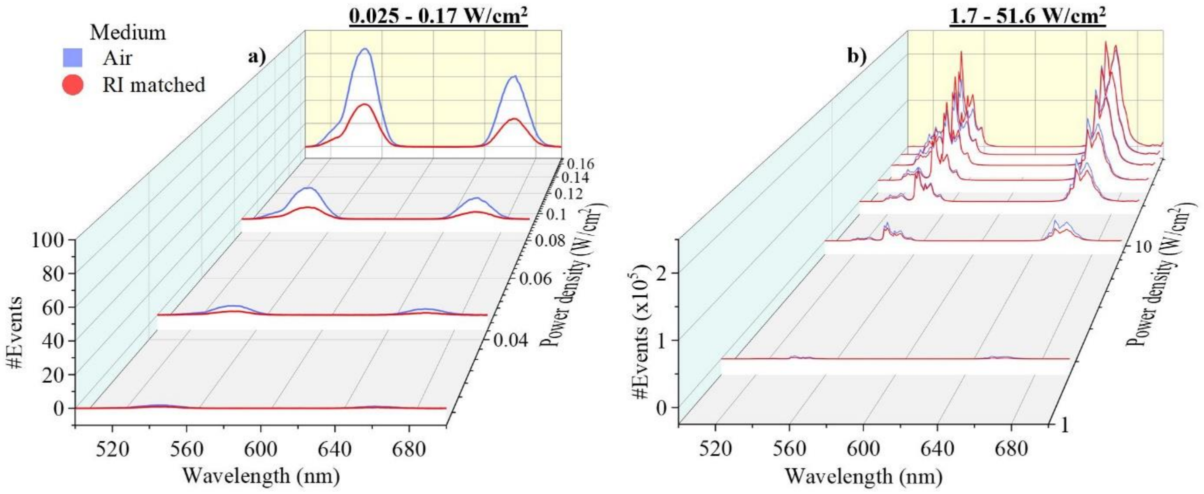

Figure S1. The emission spectra (500-700 nm) obtained for hexagonal $NaYF_4$:(18%)Yb^{3+}, (2%)Er^{3+} phosphor both in air (blue line) and in a RI-matched medium (red line) is shown over excitation PD ranges a) 0.025-0.17 W/cm^2, and b) 1.71-51.6 W/cm^2.

Supplementary note 3: Comparing reported $NaYF_4:Yb^{3+},Er^{3+}$ microscale phosphor PLQYs using review tables

From our literature review on the reported UC PLQY values and techniques used, we have found that there is a large variety in the experimental parameters that are recorded alongside the characterization. Additionally, there are a range of effects that are influencing the observed results that are rarely addressed or corrected for. To show this clearly, we have established tables (Tables. S1-2) summarizing most of the reported UC PLQY values characterizing $NaYF_4:Yb^{3+},Er^{3+}$ bulk UC materials using 980 nm excitation. The experimental parameters and influential effects associated with each value are also highlighted. Here, each effect that is known to influence the PLQY of bulk UC phosphors is alphabetically labelled as followed:

(A): Excitation beam profile. A Gaussian excitation BP has been shown to increase PD variations throughout the sample in comparison to a top-hat excitation BP [6,7].

(B): Excitation beam scattering and distortion. PD variations throughout the sample in high scattering scenarios [8,9].

(C): Primary inner-filter effect [this work]. Backscattering and absorption reduce maximum PD interacting with the sample unless the position of the excitation FP is optimized.

(D) The secondary inner-filter effect. UC emission is self-absorbed by the sample before reaching the detector [10-12]. The effect is minimized by optimizing the relationship between sample thickness and molar concentration of lanthanide dopants [10].

(E) Excitation induced thermal effects [13-15]. It is estimated that excitation induced thermal effects become significant at excitation PDs >10 W/cm^2 [14].

(F) A non-ideal sample position is used. The probability of indirect excitation increased when the sample is placed on the wall of the integrating sphere [16].

(G) Sample emission around the excitation wavelength. This leads to unreliable absorption measurements [17,18].

(H) A non-absolute PLQY method has been used.

Furthermore, the following abbreviations are used: PD: power density, BP: beam profile, Dim: dimensions, Con: concentration, V: volume, D: diameter, W; width, L: length, T: thickness, PL: path length, Em. λ: emission wavelength, Gauss: Gaussian or near-Gaussian BP, TH: Top-Hat or near-Top-Hat BP, Ellip: Elliptical BP, Ad. : influential effects that have been addressed, CPD = critical power density [14], '-': not information given, '*': information has been logically assumed by the authors, and '?': unsure on the presence of an effect due to lack of information.

Table. S1. Reviewing the UC PLQY values reported for $NaYF_4$:Yb^{3+},Er^{3+} bulk materials using 980 nm excitation, the associated experimental parameters, as well as the effects influencing each value.

Sample	Particle size<br>(shape)	Dim.<br>(Con.)	PD [W/cm^2]<br>(BP)	UCQY [%]<br>(Em. λ [nm])	Influential<br>effects	Ad.	Category<br>+ reference
β-$NaYF_4$:<br>(18%)Yb^{3+},(2%)Er^{3+}<br>in air	Several µm<br>(*Varied)	Cuboid cuvette,<br>T: 10 mm	17 ± 3<br>0.17 ± 0.03<br>0.025 ± 0.004<br>(*Gauss)	5.1 ± 0.30 (500-700)<br>0.25 ± 0.015 (500-700)<br>0.035 ± 0.0021 (500-700)	A, B, C, D, E,<br>G?	-	iPLQY<br>This work
β-$NaYF_4$:<br>(18%)Yb^{3+},(2%)Er^{3+}<br>in RI matched medium	Several µm<br>(*Varied)	Cuboid cuvette,<br>T: 10 mm	17 ± 3<br>0.17 ± 0.03<br>0.025 ± 0.004<br>(*Gauss)	2.4 ± 0.14 (500-700)<br>0.062 ± 0.0037 (500-700)<br>0.0095 ± 0.00056 (500-700)	A, B, C, D, E,<br>G?	-	iPLQY<br>This work
β-$NaYF_4$:<br>(18%)Yb^{3+},(2%)Er^{3+}<br>in air	Several µm<br>(*Varied)	Cuboid cuvette,<br>T: 10 mm	17 ± 3<br>0.17 ± 0.03<br>0.025 ± 0.004<br>(*Gauss)	5.16 ± 0.3 (500-700)<br>0.78 ± 0.046 (500-700)<br>0.13 ± 0.0079 (500-700)	A, B, C, D, E,<br>G?	C	iPLQY<br>This work
β-$NaYF_4$:<br>(18%)Yb^{3+},(2%)Er^{3+}	-<br>(*Varied)	Epoxy encapsulated<br>1×1 mm^2 glass capillary	0.7 ± 0.1<br>(*Ellip)<br><br>Saturation<br>(using CPD)	0.8 (510−542)<br>0.4 (820−870)<br><br>7.0 (*500-600)<br>(Theoretical)	A, B, C, D, G?<br><br>H	C?<br><br>-	ePLQY<br>[14]
β-$NaYF_4$:<br>(2%)Er^{3+},(18%)Yb^{3+}	-<br>(*Varied)	-	*~25	*~3.6 (*500-600)<br>*~3.2 (*600-700)<br>*~0.5 (*380-450)	H	-	-<br>[19]
$NaYF_4$:<br>(20%)Yb^{3+},(2%)Er^{3+}	4 ± 1 µm<br>(*Varied)	-	0.5 ± 0.01<br>(*Gauss)<br><br>3.8 ± 0.07<br><br>22 ± 0.44	0.21 ± 0.3 (350-900)<br>0.16 ± 0.02 (*750-900)<br>0.024 ± 0.004 (*600-700)<br>0.03 ± 0.004 (*500-600)<br><br>1.67 ± 0.3 (350-900)<br>0.86 ± 0.1 (*750-900)<br>0.43 ± 0.06 (*600-700)<br>0.37 ± 0.05 (*500-600)<br>0.014 ± 0.002 (*350-450)<br><br>7.8 ± 1.2 (350-900)<br>3.53 ± 0.50 (*750-900)<br>2.5 ± 0.4 (*600-700)<br>1.66 ± 0.2 (*500-600)<br>0.11 ± 0.02 (*350-450)	A, B, C, D, E, F,<br>G?	C?	ePLQY<br>[20]
β-$NaYF_4$:<br>(20%)Yb^{3+},(2%)Er^{3+}	*<5 µm<br>(Micro-cylindrical)	-	60<br>(*Gauss)	4.0 (*510-680)<br>1.7 (*510-580)<br>2.3 (*630-680)	A, B, C, D, E,<br>G?	C?	ePLQY<br>[21]
β-$NaYF_4$:<br>(20%)Yb^{3+},(2%)Er^{3+}	>>100 nm<br>(*Varied)	-	20<br>(*TH)	3.0 ± 0.3 (*500-600)	B, C, D, E, G?	C?	iPLQY<br>[1]
$NaYF_4$:<br>(20%)Yb^{3+},(2%)Er^{3+}	*~500 nm<br>(Hexagonal + cubic)	-	-<br>(*Gauss)	*~0.03(510-565nm)<br>*~0.04(640-680nm)<br>*~0.07 (510-680nm)	A, B, C, D, E?,<br>G?	C?	ePLQY<br>[22]
$NaYF_4$:<br>(20%)Yb^{3+},(2%)Er^{3+}	*~4500 nm<br>(Hexagonal + cubic)	-	-<br>(*Gauss)	*~4 (510-565nm)<br>*~9 (640-680nm)<br>*~13 (510-680nm)	A, B, C, D, E?,<br>G?	C?	ePLQY<br>[22]
$NaYF_4$:<br>(20%)Yb^{3+},(2%)Er^{3+}	W: 300-500,<br>L: 1900-2200 nm<br>(Cubic + hexagonal)	-	-<br>(*Gauss)	0.196 (*~500-538)<br>0.338 (*~538-580)<br>1.445 (*~620-700)<br>1.980 (*~500-700)	A, B, C, D, E?,<br>F, G?	C?	ePLQY<br>[23]
β-$NaYF_4$:<br>(21.4%)Yb^{3+},(2.2%)Er^{3+}	3 µm<br>(*Varied)	Round cuvette<br>V: 2 mm³,<br>D: 5 mm,<br>PL: 0.1 mm	20<br>(TH)<br><br>130<br>(TH)	10.3 (370-890)<br>0.92 (833-880)<br>0.13 (783-833)<br>6.4 (630-685)<br>2.4 (510-570)<br>0.17 (394-430)<br><br>8.3 (370-890)<br>0.52 (833-880)<br>0.11 (783-833)<br>5.5 (630-685)<br>1.8 (510-570)<br>0.19 (394-430)	B, D, E, G?	B	ePLQY<br>[7]

Table. S2. Reviewing the UC PLQY values reported for $NaYF_4:Yb^{3+},Er^{3+}$ bulk materials using 980 nm excitation, the associated experimental parameters, as well as the effects influencing each value.

Sample	Particle size (shape)	Dim. (Con.)	PD [W/cm^2] (BP)	UCQY [%] (Em. λ [nm])	Influential Effects	Ad.	Category + reference
$NaYF_4$: (20%)Yb^{3+},(3%)Er^{3+}	~1 µm (*Varied)	L: 8 x 8 mm T: < 1mm	21 (*Gauss)	6.9 ±0.5 (500 – 700)	A, B, C, D, E, G?	C?	ePLQY [24]
$NaYF_4$: (20%)Yb^{3+},(3%)Er^{3+}	~4.2 ± 2 µm (*Varied)	-	1 (Gauss)	0.0475 (*500-600) 0.0159 (*600-700)	A, B, C, D, G?	C?	ePLQY [8]
$NaYF_4$: Yb^{3+},Er^{3+}	- (*Varied)	D: 3mm W: 1 mm	20 (*Gauss)	2.4 (*500-600) [Converted from power conversion]	H	-	- [25]
β-$NaGdF_4$: Yb^{3+},Er^{3+}	- (*Varied)	-	*100 (*Gauss)	2.4 (*400-750)	A, B, C, D, E, G?	C?	ePLQY [26]
β-$NaYF_4$: Yb^{3+},Er^{3+} annealed at 350 °C	300 nm (Cubic)	-	50 (*Gauss)	2.5 ± 0.5 (500-700) 1.6 ± 0.3 (625-700) 0.9 ± 0.2 (500-575)	A, B, C, D, E, G?	C?	iPLQY [27]
β-$NaYF_4$: Yb^{3+},Er^{3+} annealed at 400 °C	700 nm (Deteriorated hexagonal)	-	50 (*Gauss)	4.8 ± 1 (500-700) 3 ± 0.6 (625-700) 1.8 ± 0.4 (500-575)	A, B, C, D, E, G?	C?	iPLQY [27]
β-$NaYF_4$: Yb^{3+},Er^{3+} annealed at 500 °C	2.3 µm (Deteriorated hexagonal)	-	50 (*Gauss)	2.7 ± 0.5 (500-700) 1.5 ± 0.3 (625-700) 1.3 ± 0.3 (500-575)	A, B, C, D, E, G?	C?	iPLQY [27]

Supplementary note 4: Fitted values used in the investigation of thermal effects in different scattering media and the 663 nm UC emission decay

The excitation induced thermal effects for hexagonal $NaYF_4$:(18%)Yb^{3+},(2%)Er^{3+} phosphor are investigated when the sample is in air as well as a RI-matched medium by measuring the emission decay. Equation (2) describes the function that is fitted to each decay:

$$y(x) = A1*\exp(-x/t1) + A2*\exp(-x/t2) + y0 \quad (2)$$

The 542 nm UC emission decay is presented in the main text, the average fitted values are given in Table. S3:

Table. S3 Average fitted values for the 542 nm emission decay acquired in different RI mediums.

	**Medium surrounding phosphor**	
	**Air**	**RI matched**
**y0**	5.5E+03	6.6E+03
**A1**	1.8E+03	1.3E+03
**t1**	2.3E-01	5.3E-01
**A2**	4.1E+02	5.9E+02
**t2**	1.6E+01	2.0E+01
**k1 (decay rate 1)**	4.5E+00	1.9E+00
**k2 (decay rate 2)**	6.2E-02	5.0E-02
**tau1 (half life 1)**	1.6E-01	3.7E-01
**tau2 (half life 2)**	1.1E+01	1.4E+01

Fig. S2 shows the related 663 nm UC emission decay, and Table. S4 gives the associated average fitted values.

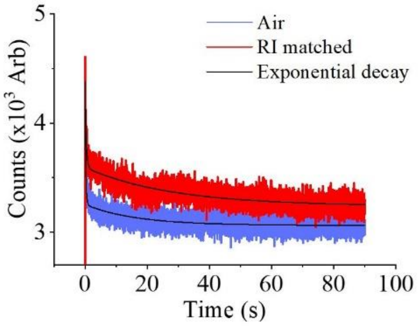

Figure S2. The 663 nm UC emission intensity vs excitation time for hexagonal $NaYF_4$:(18%)Yb^{3+},(2%)Er^{3+} in air (blue line) and a RI-matched medium (red line).

Table. S4 Average fitted values for the 663 nm emission decay acquired in different RI mediums.

	**Medium surrounding phosphor**	
	**Air**	**RI matched**
**y0**	3.1E+03	3.2E+03
**A1**	1.1E+03	6.8E+02
**t1**	1.9E-01	4.0E-01
**A2**	2.0E+02	3.1E+02
**t2**	1.6E+01	2.2E+01
**k1 (decay rate 1)**	5.5E+00	2.5E+00
**k2 (decay rate 2)**	6.4E-02	4.7E-02
**tau1 (half life 1)**	1.3E-01	2.8E-01
**tau2 (half life 2)**	1.1E+01	1.5E+01

Supplementary note 5: Simulation parameters and geometry used in COMSOL scattering investigations.

The 2D electromagnetic waves frequency domain is used for COMSOL Multiphysics 5.2a simulations. As pictured in Fig. S3, the sample media geometry is split into small rectangular sections stacked on top of each other. Parametric curves that are connected end-to-end and generated by a random Gaussian function, represent the $NaYF_4$ particles. These are distributed randomly throughout each rectangular section depending on the sample thickness required. A transverse electric wave mode is generated from Port 1 located at the bottom of the sample geometry, which represents an incident 980 nm E-field. Port 2 absorbs this E-field and possesses a perfect electrical conductor slit condition to minimize reflection. Additionally, scattering boundary conditions are placed on all other geometrical boundaries to minimize unwanted backscattering. For calculation purposes, a free triangular mesh is placed over each part of the geometry, possessing a maximum element size of 0.032 µm, and a minimum element size of 0.03 µm. Finite element analysis enabled the Poynting vector to be calculated throughout each sample, which is ultimately used to determine the power density. The imaginary RI is not considered for these simulations. The power transfer relationship between Port 1 and Port 2 is represented by the S-parameters (S21), which determines the beam transmission through each sample.

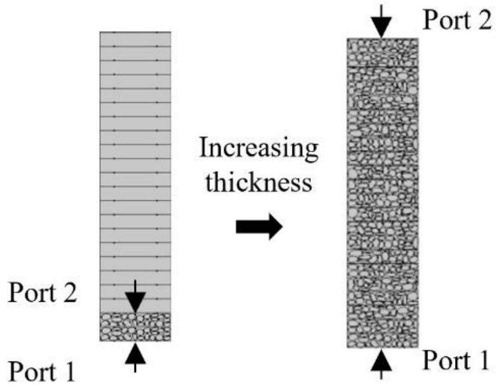

Figure S3. Images of the COMSOL simulation geometry in the thinnest (left) and thickest (right) scenarios.

Supplementary note 6: Emission spectra relating to the investigation into the influence of excitation scattering on upconversion in a low PD regime (3 x10^{-2} W/cm^2).

The Hexagonal $NaYF_4$:(18%)Yb^{3+},(2%)Er^{3+} phosphor is embedded in range of transparent solvents to obtain a variety of scattering environments. The UC emission spectra of each sample is shown in Fig. S4. Some of these RI media are obtained through mixing a ratio of chloroform (C) and toluene (T), which are miscible with each other. This is done due to the lack of transparent solvents available to create a range of media RI with small increments. The following media's are used: air (n = 1.00027 [28]), Ethanol (n = 1.3554 [29]), Hexane (n = 1.3741 [30]), Chloroform (n = 1.436 [29]), 0.8 C : 0.2 T (n = 1.44528), 0.6 C : 0.4 T (n = 1.45456), 0.4 C : 0.6 T (n = 1.46384), 0.35 C : 0.65 T (n = 1.46616), 0.3 C : 0.7 T (n = 1.46848), 0.2 C : 0.8 T (n = 1.47312), and Toluene (n = 1.4824 [29]).

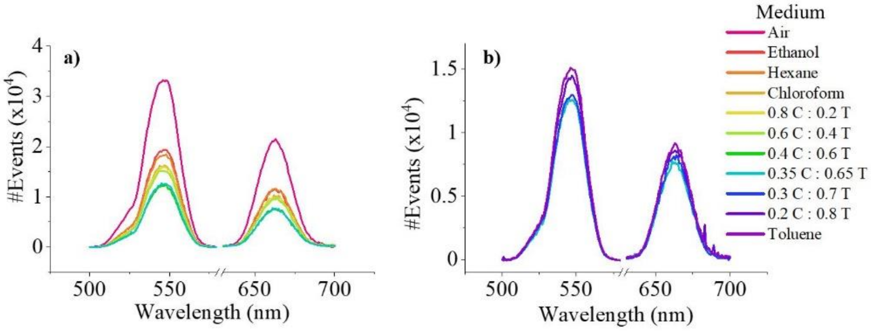

Figure S4. Hexagonal $NaYF_4$:(18%)Yb^{3+},(2%)Er^{3+} phosphor is embedded in air or a transparent medium of varying RI: air (dark pink line), Ethanol (red line), Hexane (dark orange line), Chloroform (light orange line), 0.8 C : 0.2 T (yellow line), 0.6C : 0.4 T (light green line), 0.4 C : 0.6 T (dark green line), 0.35 C : 0.65 T (light blue line), 0.3 C : 0.7 T (dark blue line), 0.2 C : 0.8 T (dark purple line), and Toluene (purple line). The emission spectra (500-700 nm) are displayed in the following RI media ranges: a) n = 1-1.466, and b) n = 1.466-1.482.

Supplementary note 7: Emission spectra relating to the investigation into the influence of the primary inner-filter effect on the observed upconversion in a high PD regime (0.169 W/cm^2).

To investigate the influence of the primary inner-filter effect, hexagonal $NaYF_4$:(18%)Yb^{3+},(2%)Er^{3+} phosphor is excited at various FP positions in the x-axis, the associated emission spectra are shown in Fig. S5. At each position, the phosphor was characterized in air, ethanol, and a RI-matched medium, to show that the effects of excitation beam scattering are also of consequence.

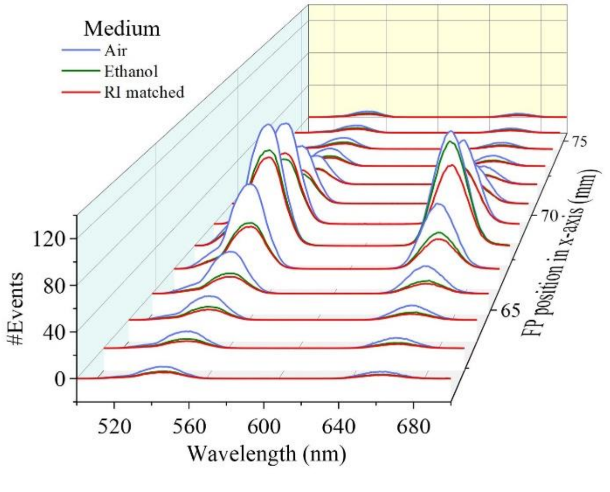

Figure S5. The emission spectra (500 – 700 nm) of hexagonal $NaYF_4$:(18%)Yb^{3+},(2%)Er^{3+} phosphor in air (blue line), ethanol (green line), or a RI matched medium (red line) is displayed, with excitation at various FP positions in the x-axis, as measured from the excitation lens.

Supplementary note 8: PLQY investigation into the influence of moving the excitation FP in the y-axis (vertically) whilst inside the sample, conducted in a high PD regime (0.169 W/cm^2).

The effect of moving the excitation FP in the y-axis (vertically) through the hexagonal $NaYF_4$:(18%)Yb^{3+},(2%)Er^{3+} phosphor is investigated, as depicted in Fig. S6(a). Pictures of the visible UC emission emitted from the phosphor at various FP positions are given in Fig. S6(b). As displayed in Fig. (c), the absorption remains constant at each FP position, as long as the light is directly incident on part of the phosphor (between the dotted lines). Similarly, no significant variation in integrated emission or ePLQY is observed in this region, as Figs. 6(d, e) show. The absorption and emission that occurs when the excitation FP is not directly incident on sample (outside of the dotted lines), is significantly reduced as the PD that the sample experiences will be far less. These values indicate the influence of partial and indirect excitation. Indirect excitation is possible because the laser light can be reflected from the integrating sphere walls and then enter the sample. However, as can be seen in Fig. S6(e) at y-axis positions 3.3 ±0.5 mm and 14.4 ±0.5, this makes minimal contribution to the ePLQY. This indicated that size ration between the sample and integrating sphere used is sufficient to minimize this effect.

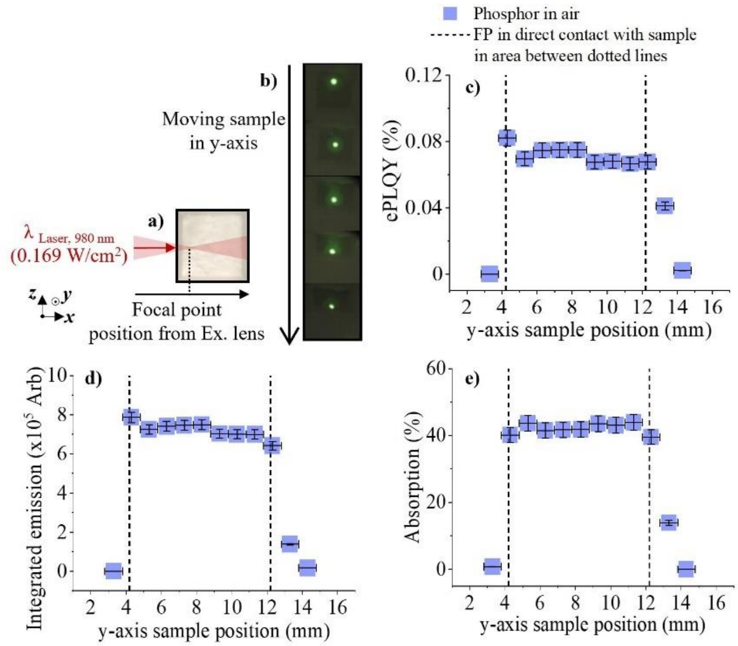

Figure S6. Hexagonal $NaYF_4$:(18%)Yb^{3+},(2%)Er^{3+} phosphor is excited with a 980 nm laser diode, the FP of which, is moved through the sample in the y-axis, as the top-down depiction in a) shows. b) Pictures of the phosphor excited at various FP positions in the y-axis. c) A depiction of how the PLQY investigation was carried out by exciting the phosphor at various y-axis sample positions relative to the cuvette holder (blue square). The following were measured against y-axis sample position: d) absorption, e) integrated emission (500-700 nm), and f) ePLQY (500-700 nm).

Supplementary note 9: Emission spectra relating to the investigation into the primary inner-filter effect on the observed upconversion in a high PD regime (0.025 – 51.6 W/cm^2).

To investigate the influence of the primary inner-filter effect in a high PD regime, hexagonal $NaYF_4$:(18%)Yb^{3+},(2%)Er^{3+} phosphor is excited at various FP positions in the x-axis. Fig. S7 displays the observed emission spectra.

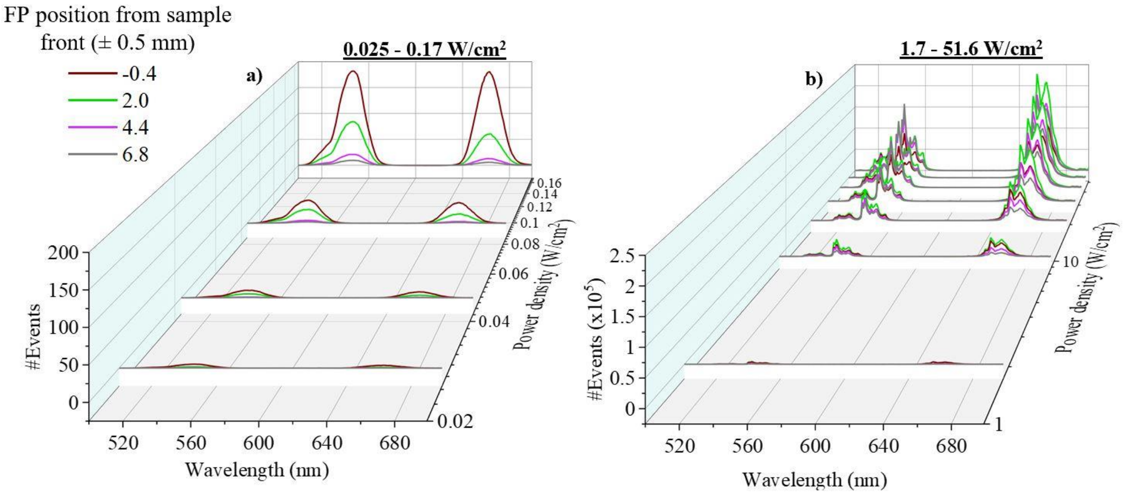

Figure S7. Hexagonal $NaYF_4$:(18%)Yb^{3+},(2%)Er^{3+} phosphor is excited at various FP positions in the x-axis. The associated emission spectra at the FP positions: -0.4 (brown line), 2 (light green line), 4.4 (pink line), and 6.8 ±0.5 mm (grey line), relative to the front of the cuvette, are presented in the following excitation PD ranges: a) 0.025 – 0.17 W/cm^2, and b) 1.71 – 51.6 W/cm^2.

Supplementary note 10: Fitted values used in the investigation of thermal effects at various excitation FP positions in the x-axis and the 663 nm UC emission decay.

Excitation induced thermal effects in hexagonal $NaYF_4$:(18%)Yb^{3+},(2%)Er^{3+} phosphor are investigated when excitation occurs at varying FP positions in the x-axis by measuring the emission decay.

The 542 nm UC emission decay is presented in the main text, the average fitted values are given in Table. S5:

Table. S5 Average fitted values for the 542 nm emission decay acquired at various excitation FP positions in the x-axis

	**Excitation FP position in the x-axis (± 0.5 mm)**			
	**-0.4**	**2**	**4.4**	**6.8**
**y0**	3.7E+03	5.5E+03	7.5E+03	7.8E+03
**A1**	1.8E+03	1.8E+03	1.5E+03	1.0E+03
**t1**	1.3E-01	2.3E-01	3.2E-01	8.1E-01
**A2**	2.8E+02	4.1E+02	5.4E+02	5.0E+02
**t2**	8.5E+00	1.6E+01	1.5E+01	2.0E+01
**k1 (decay rate 1)**	7.9E+00	4.5E+00	3.4E+00	1.3E+00
**k2 (decay rate 2)**	1.2E-01	6.2E-02	6.8E-02	4.9E-02
**tau1 (half life 1)**	9.2E-02	1.6E-01	2.2E-01	5.6E-01
**tau2 (half life 2)**	5.9E+00	1.1E+01	1.0E+01	1.4E+01

Fig. S8 shows the related 663 nm UC emission decay, and Table. S6 gives the associated average fitted values.

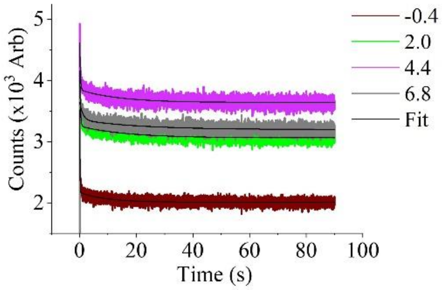

Figure S8. The 663 nm emission intensity vs excitation time for hexagonal $NaYF_4$:(18%)Yb^{3+},(2%)Er^{3+} phosphor excited at various FP positions: -0.4 (brown line), 2 (light green line), 4.4 (pink line), and 6.8 ±0.5 mm (grey line), relative to the front of the cuvette.

Table. S6 Average fitted values for the 663 nm emission decay acquired at various excitation FP positions in the x-axis

	**Excitation FP position in the x-axis (± 0.5 mm)**			
	**-0.4**	**2**	**4.4**	**6.8**
**y0**	2.0E+03	3.1E+03	3.6E+03	3.2E+03
**A1**	1.3E+03	1.1E+03	6.7E+02	3.5E+02
**t1**	9.2E-02	1.9E-01	3.2E-01	8.0E-01
**A2**	1.6E+02	2.0E+02	2.1E+02	1.7E+02
**t2**	1.1E+01	1.6E+01	1.5E+01	1.9E+01
**k1 (decay rate 1)**	1.1E+01	5.5E+00	3.3E+00	1.7E+00
**k2 (decay rate 2)**	9.6E-02	6.4E-02	6.7E-02	5.7E-02
**tau1 (half life 1)**	6.4E-02	1.3E-01	2.2E-01	5.6E-01
**tau2 (half life 2)**	7.4E+00	1.1E+01	1.0E+01	1.3E+01

Supplementary note 11: Evidence of excitation beam distortion due to exciting samples at an angle.

If a sample is excited at an angle to the incident beam, the BP will be distorted. Fig. S9(a) depicts how this type of distortion originates and its influence on the area of UC emission visible to the eye. Pictures of the hexagonal $NaYF_4$:(18%)Yb^{3+},(2%)Er^{3+} phosphor at various angles to the excitation beam are shown in Fig. S9(b). When the beam is perpendicular to the sample, the UC emission area appears circular, however, as the sample is rotated, the emission area becomes distorted.

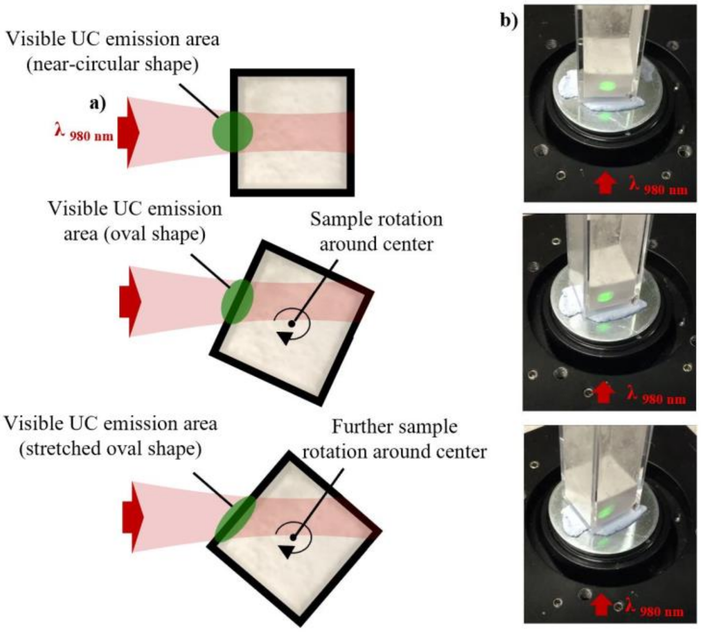

Figure S9. The hexagonal $NaYF_4$:(18%)Yb^{3+},(2%)Er^{3+} phosphor is placed on a rotating stand and excited at various angles to show the distortion in excitation BP and UC emission area, as depicted in a). b) Pictures of the phosphor's visible UC at various angles to the excitation beam.

Supplementary note 12: Emission spectra relating to the investigation into sample thickness on the observed up-conversion in a low PD regime (3 x10^{-2} W/cm^2).

The sample thickness of hexagonal $NaYF_4$:(18%)Yb^{3+},(2%)Er^{3+} phosphor is varied from 1 to 9 mm to investigate emission loss effects relating to sample geometry. The associated emission spectra are shown in Fig. S10.

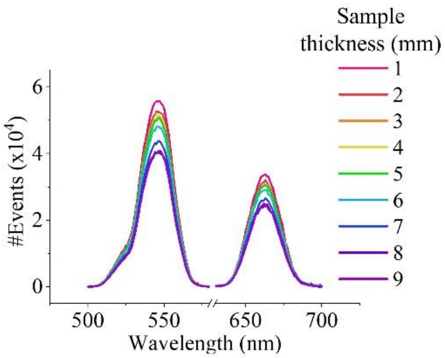

Figure S10. Emission spectra (500-700 nm) of the hexagonal $NaYF_4$:(18%)Yb^{3+},(2%)Er^{3+} phosphor samples at various sample thicknesses: 1 (pink line), 2 (red line), 3 (orange line), 4 (yellow line), 5 (green line), 6 (light blue line), 7 (dark blue line), 8 (dark purple line), and 9 (purple line) ±0.5 mm.

As described in the main text, the ePLQY and integrated emission are also determined. These are fitted using equation (3) and the values displayed in Table. S7 are determined.

$$y = a + b*x \quad (3)$$

Table. S7 Fitted values for the ePLQY and integrated emission acquired from characterizing various thickness of UC phosphor

	**iPLQY**	**Integrated emission**
**Intercept**	3.9E-02 ± 7.5E-04	2.5E+06 ± 5.5E+04
**Slope**	-1.2E-03 ± 1.2E-04	-8.1E+04 ± 9.0E+03
**Adj. R-Square**	0.91927	0.90939

Supplementary note 13: Estimating the magnitude of UC emission self-absorption.

A self-absorption estimation is determined by measuring the magnitude of 540 nm and 660 nm Xe lamp light absorption by the hexagonal $NaYF_4$:(18%)Yb^{3+},(2%)Er^{3+} phosphor in comparison to its undoped counterpart. As Fig. S11 shows, the straight beam spectra are measured in the integrating sphere after the beam has passed through either the doped or undoped samples. By subtracting these spectra, the absorption of 540 nm and 660 nm light by the doped sample is obtained, and thus an estimation as to how much UC emission is self-absorbed during our experiments in the range 500-700 nm is determined.

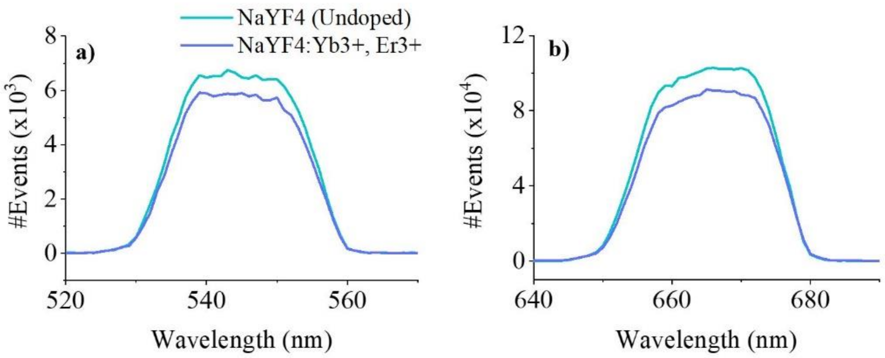

Figure S11. Xe lamp light is incident on the 9 mm thick hexagonal $NaYF_4$:(18%)Yb^{3+},(2%)Er^{3+} phosphor (dark blue line), or its undoped counterpart (light blue line), inside the integrating sphere. The straight beam spectra are measured for incident light centered at a) 540 nm, and b) 660 nm.

Supplementary note 14: Estimating the magnitude of UC emission loss due to backscattering (light leakage).

The estimation for light leakage due to backscatter out of the integrating sphere is determined by measuring the magnitude of 540 nm and 660 nm Xe lamp light after propagating through the undoped phosphor in comparison to an empty cuvette. The straight beam spectra, shown in Fig. S12, are measured inside the integrating sphere. By subtracting these spectra, an estimation is determined regarding how much light (500-700 nm) is lost out of the integrating sphere due to backscattering from the powder sample.

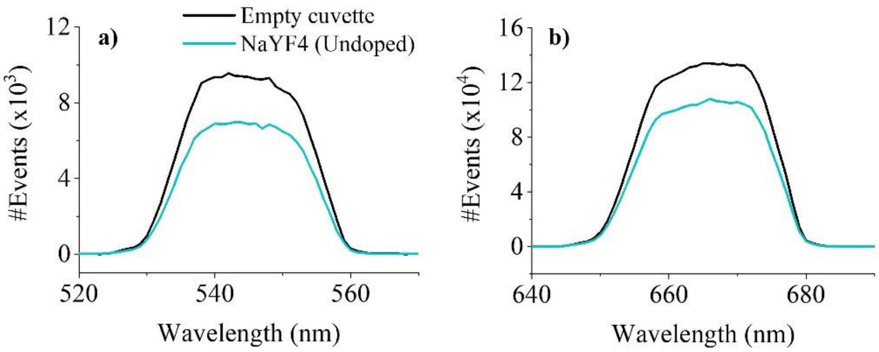

Figure S12. Xe lamp light is incident on the 9 mm thick undoped phosphor (light blue line), or an empty cuvette (black line), inside the integrating sphere. The straight beam spectra are measured for incident light centered at a) 540 nm, and b) 660 nm.

Supplementary note 15: Emission spectra relating to the investigation into the influence of cuvette type on the observed upconversion in a high PD regime (0.169 W/cm^2).

To investigate the influence of cuvette type on the observed UC, hexagonal $NaYF_4$:(18%)Yb^{3+},(2%)Er^{3+}phosphor is embedded in a cuboid cuvette and a cylindrical vial. The emission spectra are measured at various FP positions in the x-axis to compare the maximum intensities possible, as shown in Fig. S13 (a, b).

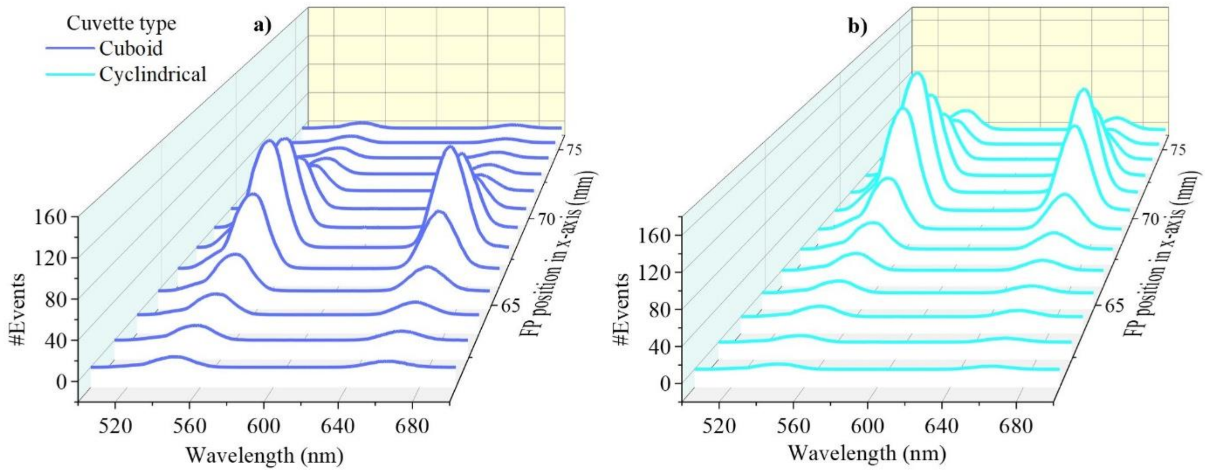

Figure S13. The emission spectra (500 – 700 nm) of hexagonal $NaYF_4$:(18%)Yb^{3+},(2%)Er^{3+} phosphor excited a various FP positions in the x-axis when in a) a cuboid cuvette (dark blue line), and b) a cylindrical vial (light blue line).

Supplementary note 16: Simulation parameters and geometry used in Zemax investigations.

The simulation geometry is implemented in Zemax™ optical design software (Version 20.1) and has resemblance to the FLS920 spectrophotometer (Edinburgh Instruments) optical setup used experimentally throughout this work. The light (λ = 980 nm) originates from an on-axis point source. The back surface of a lens of focal length f = 115 mm, thickness d = 8 mm, n = 1.45, and diameter D = 50 mm is located 210.6 mm away from the point source. An identical but inverted lens is located 0.6 mm after the first lens. The sample is placed 61 - 66 mm away from the back of the second lens depending on the configuration. A numerical aperture of 0.103 in the object space is used for all the simulations. A similar RI (n = 1.4507) and glass thickness (1 mm) is used for both vials. Furthermore, a RI of n = 1.466 is used for the material inside each vial. The round vial is modelled using an outer curved surface of radius R = 3 mm and inner curved surface of radius R = 2 mm. Pictures of the simulation geometry can be seen in Fig. S14 (a, b). In these instances, the optimal distance between the second lens and the vial is used for the focus to occur 2 mm inside the vials (after the inner surfaces). The simulated BPs at the optimal FP inside the square cuvette and circular vial are shown in Fig. S14(c) as the distance between the sample and excitation lens is varied.

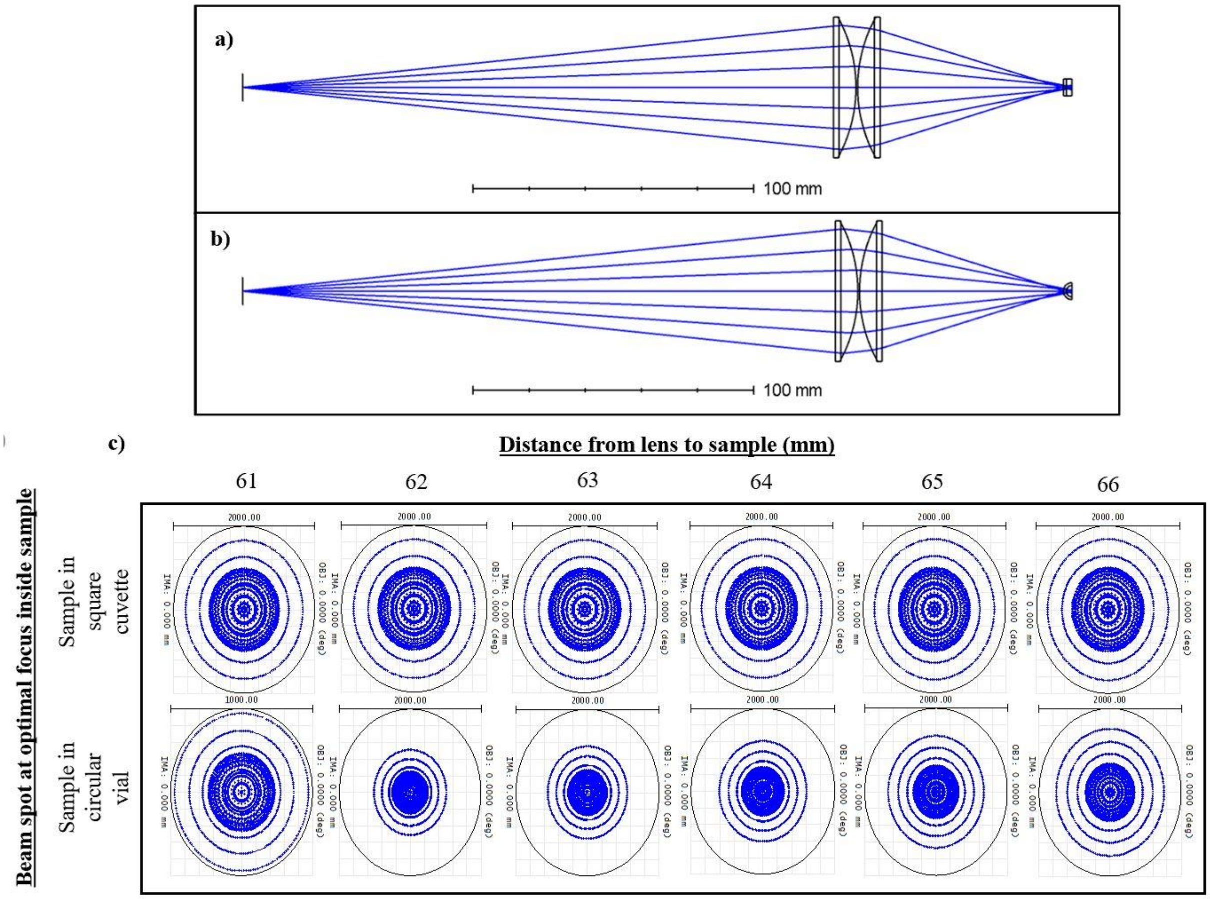

Figure S14. Using Zemax, simulations are generated that focus 980 nm light into either a square cuvette or circular vial. The geometry is given for the simulations regarding the a) square cuvette, and b) circular vial. The samples are moved closer to the excitation lens to replicate different laser penetration depths. In doing so, the BP at the position of optimal focus inside each sample is found at different laser penetration depths, as shown in c).

Supplementary note 17: Synthesis of $NaYF_4$:(18%)Yb^{3+},(2%)Er^{3+} UC phosphor.

Stoichiometric amounts of Y_2O_3, Yb_2O_3, and Er_2O_3 (6N, Metall Rare Earth Ltd.) were dissolved in HNO_3 (65%, Merck KGaA) in a Teflon beaker on a sand bath. The acid was evaporated, and HF acid (40%, Merck KGaA) added three times. Na_2CO_3 (4N7, AlfaAesar) was dissolved in water and added to the rare earth solution to obtain a Na/rare-earth ratio of 2/1. The solution was evaporated, and HF acid added two more times. Then the solution was dried up, the powder ground in an agate mortar, and transferred into a glassy carbon boat for fluorination. The powder was heated in an alloy 600 apparatus in a HF/Ar gas flow to 550°C for 20 hours, ground, and heated in an Ar gas flow to 590°C for another 20 hours. Finally, excess NaF was washed off in water and the product dried at 80°C in air. Phase purity was verified by powder X-ray diffraction. The product consists of few µm sized crystallites according to electron microscopy. As seen in Fig. S15, both the undoped and doped powders consisted of similar sized particles

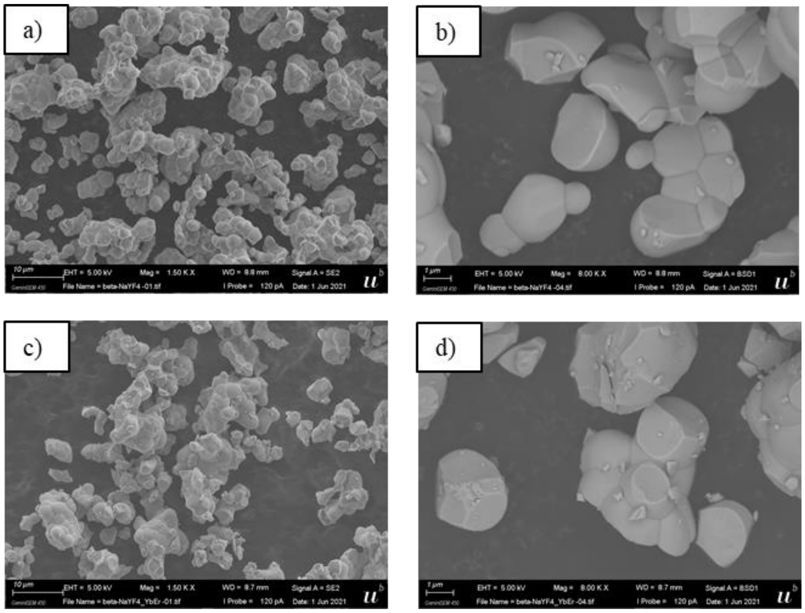

Figure S15. SEMs of the hexagonal $NaYF_4$:(18%)Yb^{3+},(2%)Er^{3+} phosphor at a) 1500x magnification and b) 8000x magnification. SEMs of the undoped $NaYF_4$ reference sample at c) 1500x and d) 8000x magnification.

Supplementary note 18: Experimental characterization methodology (PLQY and thermal investigations).

PLQY experimental method for the scattering investigation in the high PD regime. The PLQY is characterized for the following UC transitions; $^2H_{11/2}/^4S_{3/2} \rightarrow {}^4I_{15/2}$ and $^4F_{9/2} \rightarrow {}^4I_{15/2}$, over the range 500-700 nm [31]. A 980 nm laser diode (LSR980NL-3W, by Lasever Inc.) was used to achieve excitation in a high PD regime. An average spot size of 5.8 x10^{-3} cm^2 was recorded with an error of 16%, by conducting three measurements using the razor blade method. The laser driver was kept constant to minimise thermal drift and power instability issues, and the PD was altered over the range 0.025 – 51.6 W/cm^2 by using an optical density wheel. The emission monochromator slits were 20 nm wide for PDs below 0.169 W/cm^2, and 0.2 nm wide for PDs above this PD value. This was necessary to protect the detectors from oversaturation at high PDs, whilst still enabling sufficient emission intensity to be recorded at low PDs. To be comparable, the integrated emission was corrected for using a slit correction factor that was obtained by measuring the sample emission at an appreciable PD (0.48 W/cm^2) using both emission slits. The step size was 1 nm for emission scans. For straight beam measurements, the step size was 0.1 nm or 1 nm depending on if the emission slits were 0.2 nm or 20 nm, respectively. The PLQY measurements were unaffected by this change in measurement settings. The PLQY data represents the average after measuring three times. The same phosphor sample was measured ten times at a constant PD to achieve a standard deviation that was used to apply error bars to each data point. This was done to be more inclusive of errors that originated from the sample's position, as well as those arising from laser instability, excitation induced thermal effects, and the measurement equipment.

Investigation into excitation induced thermal effects. These investigations were conducted using 980 nm laser excitation at a PD of 51.6 W/cm^2. The emission slits remained constant at 20 nm wide. Prior to excitation, a kinetic scan of the sample would be initiated, which took either 542 or 663 nm emission intensity measurements every 0.01 sec. Within the first few seconds of the scan, a mechanical door would be lifted allowing for the laser to interact with the sample, and the decrease in emission to be recorded over a 90 sec period. Average values are reported by measuring each sample three times. The standard deviation was used to apply error bars.

PLQY experimental method for the scattering investigation in the low PD regime. The Xe lamp used in this section was found to have a rectangular BP and a spot size of 0.241 cm^2, with an error of 3%. This was determined through three measurements using the razor blade technique and equated to an excitation PD of 3 x10^{-2} ± 1 x10^{-3} W/cm^2. The Xe lamp was centred to 980 nm and the excitation monochromator slits were 22 nm wide. Furthermore, the emission monochromator slits were 20 nm wide and a 610 long-pass filter was placed in the light path to minimise non-linear effects. Each data point was measured three times and the average is reported. The same sample was measured ten times to find a standard deviation, which was used to apply error bars to each data point. The ePLQY was presented so that the result indicated the RI of the doped phosphor only.

PLQY experimental method for primary inner-filter effect investigation. These measurements were conducted with a 980 nm laser diode at a constant PD of 0.169 W/cm^2. The emission slits remained at 20 nm wide throughout. A ruler was used to measure the various FP positions, which were achieved by adjusting the excitation lens. The error in FP position was ±0.5 mm. The spectral data was repeated three times and the average is reported. The error bars applied to each data point originated from the previously discussed errors established for laser diode measurements. Similar parameters, methodology, and errors were also used later to characterize the phosphor in different cuvette types at various FP positions. The 10 x 10 mm cuboid cuvette possessed a 1.2 mm thick wall diameter made from quartz glass, and the cylindrical cuvette had a 0.3 mm radius with a 0.1 mm thick wall made from borosilicate glass.

PLQY experimental method for sample geometry investigation. To ensure each sample had a similar height, they were prepared using a ratio (1 : 0.4) of sample thickness (mm) to sample weight (g). The sample thicknesses were enforced using a home-made transparent wall separator, which was cut out of a soda lime glass microscope slide using a diamond pen. The edges were then smoothed using diamond encrusted sandpaper. This material was chosen due to its high transmission in the visible-NIR region. It was ensured that all samples possessed one glass wall to make the results comparable. The thickness of the samples was measured using standard calipers. For PLQY measurements, the Xe lamp was used as the source, with an identical PD and monochromator slits to those previously mentioned. The error bars applied to each data point originated from the previously discussed errors established for Xe lamp measurements.

References

1 Boyer, J. C. & van Veggel, F. C. Absolute quantum yield measurements of colloidal NaYF4: Er3+, Yb3+ upconverting nanoparticles. *Nanoscale* **2**, 1417-1419, doi:10.1039/c0nr00253d (2010).

2 de Mello, J. C., Wittmann, H. F. & Friend, R. H. An improved experimental determination of external photoluminescence quantum efficiency. *Advanced materials* **9**, 230-232 (1997).

3 Faulkner, D. O. *et al.* Measurement of absolute photoluminescence quantum yields using integrating spheres–Which way to go? *Laser & Photonics Reviews* **6**, 802-806 (2012).

4 Meijer, M. S. *et al.* Absolute upconversion quantum yields of blue-emitting LiYF4:Yb(3+),Tm(3+) upconverting nanoparticles. *Physical Chemistry Chemical Physics* **20**, 22556-22562, doi:10.1039/c8cp03935f (2018).

5 Gao, G. *et al.* Finely-tuned NIR-to-visible up-conversion in La 2 O 3: Yb 3+, Er 3+ microcrystals with high quantum yield. *Journal of Materials Chemistry C* **5**, 11010-11017 (2017).

6 Mousavi, M. *et al.* Beam-profile-compensated quantum yield measurements of upconverting nanoparticles. *Physical Chemistry Chemical Physics* **19**, 22016-22022 (2017).

7 Kaiser, M. *et al.* Power-dependent upconversion quantum yield of NaYF 4: Yb 3+, Er 3+ nano-and micrometer-sized particles–measurements and simulations. *Nanoscale* **9**, 10051-10058 (2017).

8 Jones, C. M. *et al.* Effect of light scattering on upconversion photoluminescence quantum yield in microscale-to-nanoscale materials. *Optics express* **28**, 22803-22818 (2020).

9 Jones, C. M., Wang, X. & Marques-Hueso, J. in *3D Image Acquisition and Display: Technology, Perception and Applications.* JW5C. 4 (Optical Society of America).

10 Boccolini, A., Marques-Hueso, J. & Richards, B. S. Self-absorption in upconverter luminescent layers: impact on quantum yield measurements and on designing optimized photovoltaic devices. *Optics letters* **39**, 2904-2907 (2014).

11 Boccolini, A., Favilla, E., Tonelli, M., Richards, B. S. & Thomson, R. R. Highly efficient upconversion in Er 3+ doped BaY 2 F 8 single crystals: dependence of quantum yield on excitation wavelength and thickness. *Optics Express* **23**, A903-A915 (2015).

12 Tan, M. C., Al-Baroudi, L. & Riman, R. E. Surfactant effects on efficiency enhancement of infrared-to-visible upconversion emissions of NaYF4: Yb-Er. *ACS applied materials & interfaces* **3**, 3910-3915 (2011).

13 Joseph, R. E. *et al.* A method for correcting the excitation power density dependence of upconversion emission due to laser-induced heating. *Optical materials* **82**, 65-70 (2018).

14 Joseph, R. E. *et al.* Critical Power Density: A Metric To Compare the Excitation Power Density Dependence of Photon Upconversion in Different Inorganic Host Materials. *The Journal of Physical Chemistry A* **123**, 6799-6811 (2019).

15 Würth, C. *et al.* Excitation power dependent population pathways and absolute quantum yields of upconversion nanoparticles in different solvents. *Nanoscale* **9**, 4283-4294 (2017).

16 Pollnau, M. Power dependence of upconversion luminescence in lanthanide and transition-metal-ion systems. *Physical Review B* **61**, 3337 (2000).

17 Fischer, S., Bronstein, N. D., Swabeck, J. K., Chan, E. M. & Alivisatos, A. P. Precise tuning of surface quenching for luminescence enhancement in core–shell lanthanide-doped nanocrystals. *Nano letters* **16**, 7241-7247 (2016).

18 Fischer, S. *et al.* Small alkaline-earth-based core/shell nanoparticles for efficient upconversion. *Nano letters* **19**, 3878-3885 (2019).

19 Anderson, R. B., Smith, S. J., May, P. S. & Berry, M. T. Revisiting the NIR-to-visible upconversion mechanism in β-NaYF4: Yb3+, Er3+. *The journal of physical chemistry letters* **5**, 36-42 (2014).

20 Pokhrel, M., kumar Gangadharan, A. & Sardar, D. K. High upconversion quantum yield at low pump threshold in Er3+/Yb3+ doped La2O2S phosphor. *Materials Letters* **99**, 86-89 (2013).

21 Fan, S. *et al.* Absolute up-conversion quantum efficiency reaching 4% in β-NaYF 4: Yb 3+, Er 3+ micro-cylinders achieved by Li+/Na+ ion-exchange. *Journal of Materials Chemistry C* **6**, 5453-5461 (2018).

22 Faulkner, D. O., Petrov, S., Perovic, D. D., Kherani, N. P. & Ozin, G. A. Absolute quantum yields in NaYF4: Er, Yb upconverters–synthesis temperature and power dependence. *Journal of Materials Chemistry* **22**, 24330-24334 (2012).

23 Qin, H. *et al.* Tuning the upconversion photoluminescence lifetimes of NaYF 4: Yb 3+, Er 3+ through lanthanide Gd 3+ doping. *Scientific reports* **8**, 1-8 (2018).

24 Stanton, I. N. *et al.* Power-dependent radiant flux and absolute quantum yields of upconversion nanocrystals under continuous and pulsed excitation. *The Journal of Physical Chemistry C* **122**, 252-259 (2018).
25 Page, R. H. *et al.* in *Advanced solid state lasers.* VL3 (Optical Society of America).
26 Li, X. *et al.* Successive layer-by-layer strategy for multi-shell epitaxial growth: shell thickness and doping position dependence in upconverting optical properties. *Chemistry of Materials* **25**, 106-112 (2013).
27 Dyck, N. C., Van Veggel, F. C. & Demopoulos, G. P. Size-dependent maximization of upconversion efficiency of citrate-stabilized β-phase NaYF4: Yb3+, Er3+ crystals via annealing. *ACS applied materials & interfaces* **5**, 11661-11667 (2013).
28 Ciddor, P. E. Refractive index of air: new equations for the visible and near infrared. *Applied optics* **35**, 1566-1573 (1996).
29 Kedenburg, S., Vieweg, M., Gissibl, T. & Giessen, H. Linear refractive index and absorption measurements of nonlinear optical liquids in the visible and near-infrared spectral region. *Optical Materials Express* **2**, 1588-1611 (2012).
30 Kozma, I. Z., Krok, P. & Riedle, E. Direct measurement of the group-velocity mismatch and derivation of the refractive-index dispersion for a variety of solvents in the ultraviolet. *JOSA B* **22**, 1479-1485 (2005).
31 Homann, C. *et al.* NaYF4: Yb, Er/NaYF4 core/shell nanocrystals with high upconversion luminescence quantum yield. *Angewandte Chemie International Edition* **57**, 8765-8769 (2018).
